# Hsp90-downregulation influences the heat-shock response, innate immune response and onset of oocyte development in nematodes

**DOI:** 10.1371/journal.pone.0186386

**Published:** 2017-10-27

**Authors:** Julia Eckl, Siyuan Sima, Katrin Marcus, Claudia Lindemann, Klaus Richter

**Affiliations:** 1 Center for Integrated Protein Science at the Technische Universität München, Department Chemie, Lichtenbergstr.Garching, Germany; 2 Ruhr-University Bochum, Medizinisches Proteom-Center, Functional Proteomics, Universitätsstrasse, Bochum, Germany; Université de Genève, SWITZERLAND

## Abstract

Hsp90 is a molecular chaperone involved in the regulation and maturation of kinases and transcription factors. In *Caenorhabditis elegans*, it contributes to the development of fertility, maintenance of muscle structure, the regulation of heat-shock response and *dauer* state. To understand the consequences of Hsp90-depletion, we studied Hsp90 RNAi-treated nematodes by DNA microarrays and mass spectrometry. We find that upon development of phenotypes the levels of chaperones and Hsp90 cofactors are increased, while specific proteins related to the innate immune response are depleted. In microarrays, we further find many differentially expressed genes related to gonad and larval development. These genes form an expression cluster that is regulated independently from the immune response implying separate pathways of Hsp90-involvement. Using fluorescent reporter strains for the differentially expressed immune response genes *skr-5*, *dod-24* and *clec-60* we observe that their activity in intestinal tissues is influenced by Hsp90-depletion. Instead, effects on the development are evident in both gonad arms. After Hsp90-depletion, changes can be observed in early embryos and adults containing fluorescence-tagged versions of SEPA-1, CAV-1 or PUD-1, all of which are downregulated after Hsp90-depletion. Our observations identify molecular events for Hsp90-RNAi induced phenotypes during development and immune responses, which may help to separately investigate independent Hsp90-influenced processes that are relevant during the nematode’s life and development.

## Introduction

Hsp90 is an ATP-dependent molecular chaperone conserved from bacteria to mammals. The cytosolic version of Hsp90 is essential in all eukaryotes. In the nematode *C*. *elegans* the Hsp90-homologue DAF-21 is required for development [1–3]. This is evident from the complex phenotype that results from RNA interference (RNAi) against Hsp90, which combines features of developmental misregulation during gonad development, muscle structure organization and vulva development. The nematodes arrest in a later larval stage and often lack one of the two gonad arms [4]. Furthermore, embryo development is disrupted and endomitotic oocytes are formed [1, 4, 5]. Beyond these phenotypes, Hsp90 guarantees the balanced state of proteostasis under normal growth conditions [5–8]. Hsp90 is also involved in the regulation of the *dauer* state, as the E292K variant of DAF-21 induces a constitutive entry into this stress-resistant condition [1, 9, 10]. Regarding its cellular function, Hsp90 is thought to support various client proteins during their maturation process, including many protein kinases and several transcription factors [11]. Additionally, the large number of cofactors adding substrate specificity to Hsp90 implies that even more clients can be processed with the help of the Hsp90-cofactors FKB-6, STI-1, UNC-45, PPH-5 and the Xap2-homolog AIPR-1/C56C10.10. Additional cofactors have been show to link Hsp90 to vesicle control (TTC1, C34B2.5 in *C*.*elegans*), transcription (RPAP3, C33H5.8 in *C*.*elegans*), mitochondrial import (Tom70, *C*. *elegans* homolog ZK370.8) and ribosomal functions (SGT-1 and the Cns1p-homolog C17G10.2) [12–17].

Thus, reducing the levels of Hsp90 by RNAi may interfere with several pathways making it challenging to disentangle the events that ultimately lead to arrest of development. Understanding activities at specific pathways is further complicated by the observation that few Hsp90-cofactors induce an obvious phenotype upon RNAi knock down. The stronger effects relate to delay in larval development upon depletion of the kinase-specific cofactor CDC-37 [18–20]. RNAi against the myosin-interacting cofactor UNC-45 results in sterility and disorganization of body wall muscle sarcomeres and the paralysis of the nematodes [5, 21]. But for most Hsp90-cofactors, no morphological changes have been observed upon RNAi knock-down. Therefore improved techniques and systems must be employed to untangle the activities each cofactor plays.

By using nematode strains containing p*hsp-16*.*2*::*GFP* and p*hsp-70*::*GFP*, *w*e previously had observed that Hsp90-depletion leads to an induction of the heat-shock response in body wall muscle and intestinal cells [8, 15]. Here we aim to identify further reporter strains to help better define changes that occur after Hsp90-depletion. To this end, we employ mass spectrometry and DNA microarrays on Hsp90-depleted nematodes at the onset of phenotype development and then investigate prominent differentially expressed genes.

## Materials and methods

### Strains and growth

Nematodes were handled according to established protocols [22]. For normal growth, nematodes were cultivated on NGM plates and fed with OP50 bacteria at 20 °C. During RNAi experiments nematodes were maintained on NGM plates supplemented with ampicillin, tetracycline and 1 mM IPTG. HT115 (DE3) bacteria were transformed with the plasmids L4440 or L4440-*daf-21* [5, 23] and were grown in 5 ml LB_Amp,Tet_. At an OD_600_ of 0.6 dsRNA expression in these cultures was induced with 1 mM IPTG for four hours. After that the dsRNA-expressing bacteria were placed on RNAi plates and synchronized L1 nematodes–prepared by bleaching and hatched overnight in M9-buffer–were added onto the plates with induced bacteria. RNAi experiments were performed at 20 °C until phenotypes became obvious after 2.5 days. Nematodes were then harvested for proteomic or transcriptomic analysis or for imaging. Small heterogeneity in RNAi-treated nematodes was observed at the time of harvesting in different biological replicates. Thus control nematodes were cultivated for one further day to confirm the full development of the expected phenotypes.

### Mass spectrometric analysis

To perform a mass spectrometric analysis the RNAi treatment was performed on plates made from bacterial M9-medium supplemented with ampicillin, tetracycline and 1 mM IPTG. This bacterial growth medium contains 2 g/L glucose and 1 g/L NH_4_SO_4_ as the carbon and nitrogen sources [24, 25]. To label the nematodes with ^15^N, 1 g/L ^15^NH_4_SO_4_ (euriso-top GmbH, Saarbrücken, Germany) was used instead of the normal ^14^NH_4_SO_4_. Nematode growth was not affected by the ^15^N-containing nitrogen source. Nematodes were harvested after 2.5 days by washing them off the plates with M9-buffer. They were separated from residual bacteria by gravity sedimentation in a 15 mL plastic tube. The supernatant was removed and after three such washing steps the nematodes were lysed in a Retsch Mixer Mill MM400 in 1 mL M9-buffer. The lysate was cleared by centrifugation and the protein concentration was determined by Bradford reagent. The isotope-tagged lysates were then mixed at the calculated ratio to ensure that equal amounts of each sample were combined. This sample was frozen and stored at -80 °C until mass spectrometric analysis was performed.

After a tryptic digest of the proteins, 15 μL of the sample peptides were analyzed by nanoLC-MS/MS on an UltiMate® 3000 UPLC system (Thermo Fisher Scientific) online coupled to a Q Exactive Hybrid-Quadropole-Orbitrap mass spectrometer (Thermo Fisher Scientific) with electrospray ion source (ESI). Initially, the samples were desalted and concentrated on a 75 μm x 20 mm Acclaim® PepMap100 C18 column with 5 μm particle size (Thermo Fisher Scientific) with a flow rate of 30 μL/min using 95% solvent A (0.1% TFA in H_2_O_dest_) and 5% solvent B (0.1% TFA, 50% ACN in H_2_O_dest_) for 7 minutes. Peptides were subsequently loaded onto a 75 μm x 50 cm Acclaim® PepMap100 RSLC C18 column with 2 μm particle size (Thermo Fisher Scientific) using a mixture of 95% solvent C (0.1% formic acid (FA) in H_2_O_dest_) and 5% solvent D (0.1% FA, 84% ACN in H_2_O_dest_). The elution of peptides was performed at a constant flow rate of 400 nL/min in a linear gradient of 5% to 40% solvent D over 120 minutes. Mass spectrometry was performed in the data-dependent acquisition (DDA) mode with Full scan Fourier transform mass spectrometry (FTMS) acquired in an m/z range of 350 to 1400 with a resolution of 70,000. The ten most intense peptides (charge range ≥ +2) in the FTMS-scan were selected for higher-energy collisional dissociation (HCD) with the collision energy (NCE) set to 27. The dynamic exclusion time was set on 30 seconds with an MS/MS resolution of 35.000 within an m/z range of 300 to 2000. Data analysis was performed with Mascot Distiller (Matrix Science Inc., USA) as described [25] using a Uniprot database for *C*. *elegans*. The mass spectrometry proteomics data have been deposited to the ProteomeXchange Consortium via the PRIDE partner repository with the dataset identifier PXD006185 and 10.6019/PXD006185 [PubMed ID: 26527722].

### Microarray sample preparation and handling

After RNAi treatment for roughly 2.5 days nematodes were harvested for DNA microarray experiments. Hsp90-depleted and control vector treated nematodes were washed off the plates and collected in 15 mL plastic tubes. Nematodes were separated from residual bacteria by gravity sedimentation. The worms were frozen at -80 °C. RNA extraction and analysis of the mRNA levels was performed at the Kompetenzzentrum für Fluoreszente Bioanalytik (KFB) as a for-fee service. For this analysis Affymetrix GeneChip *C*. *elegans* Genome Arrays were used and the data for all genes were RMA-normalized. All microarray datasets will be uploaded to the GEO repository.

### Network calculation and evaluation

To compare expression values under various conditions and to identify the most prominent hits we used Microsoft Excel and the clustering program ClusterEx, which was implemented by us to perform semi-quantitative gene clustering based on correlations found in preformed coexpression databases [26, 27]. To obtain the initial hit list the differential expression values from the three biological replicates were averaged to yield the mean expression difference and the standard deviations for each gene. The 250 strongest affected genes for each direction were retained and non-protein coding genes were excluded. To construct networks we obtained the 40 highest-ranking coregulated partner genes for each hit from a *C*. *elegans* coexpression database obtained from http://seek.princeton.edu/modSeek/worm [28]. Coexpression counts for the hit genes were collected by ClusterEx from this database [26], adding 41x41 connections per included hit gene, leading to network matrices composed of 420250 connections for 250 genes. In network calculations based on real expression data most hits were connected to many other hit genes. This was not the case for random control groups of genes, where few hit-to-hit pairs were generated, but for most genes no coexpressed hit was included. To test the quality of the clusters in the networks, further genes, which had most connections to hit genes, were determined, integrated into the networks (marked with a pink border) and evaluated to confirm the predictive accuracy (CoRegScore) of the network/database setup. This procedure was automated in ClusterEx as described [26]: In short, all genes were ranked according to their expression changes and the positions of the predicted genes were determined and summed up. This value then was used to calculate the CoRegScore between 100 and -100 as described [26]: In the following equation the sum of the predicted genes (AddedRealPositions) is set in relation to the best possible prediction (AddedIdealPositions) and the worst possible prediction (AddedWorstPositions).

$$CoRegScore=100-200*\frac{AddedRealPositions-AddedIdealPositions}{AddedWorstPositions-AddedIdealPositions}$$

For statistical analysis random controls were determined. To this end 100 similar-sized random lists of genes were generated and the analysis was performed in an identical manner. Based on the resulting mean and standard deviation, the significance/p-value for each parameter was determined by calculating the Z-score (http://www.socscistatistics.com/tests/ztest/zscorecalculator.aspx) and using a one-tailed hypothesis at http://www.socscistatistics.com/pvalues/normaldistribution.aspx. For all networks in this study the connection parameters show a high level of significance compared to random gene lists (p<0.001).

To test the influence of the specific database, we performed identical calculations with coexpression relationships downloaded from SPELL (http://spell.caltech.edu:3000/) [29] or from COXPRESdb [30] and qualitatively similar networks were obtained. To allow other users to use the described clustering approach for their own *C*. *elegans* expression data, we will include it into our webserver at www.clusterex.de [26].

### Network visualization and layouting

Networks were finally visualized in CytoScape based on the ClusterEx output files containing all the hit-to-hit connections and their occurrence counts. The final layout was based on the “Edge-weighted Spring Embedded Layout”, which clusters the genes according to their connections and brings highly connected genes close to each other [31, 32]. Individual nodes were moved only to prevent overlapping nodes in plot areas, where clustering was very high, but care was taken not to alter the location relative to neighboring nodes.

Network construction for the mass spectrometry samples was also performed with ClusterEx. As no database reporting on coexpression at the protein level was available to us, we also used the modSEEK transcriptional coexpression database here. To compensate for the very small number of hits and the many proteins, for which we did not obtain quantitative expression values in the mass spectrometry experiment, we used the top 100 coregulated genes to construct the networks, instead of 40 as for the microarray data.

### Comparison of different microarray experiments regarding similarities

Data from our arrays were compared to publicly available experiments investigating RNAi against *sbp-1* and *sams-1* (GSM1816551-GSM1816559) [33]. The corresponding CEL-files were obtained from the GEO repository [34]. Expression values were extracted and normalized with RMADataConv and RMAExpress [35]. Similarly we obtained the CEL-files and result files for the response of N2 nematodes to pathogenic bacteria, in particular to the *Vibrio cholerae* strains VC109 and VC110 (GSE34026) [36] and to *Pseudomonas aeruginosa* (GSE5793) [37, 38]. To visualize the similarity to the Hsp90 RNAi response, the expression values for the Hsp90-RNAi responsive genes were retrieved from the experiments and then used to color the Hsp90-depletion response according to the respective data. The same color code was used in the supplemental figures to allow a comparison of the same genes within the visualized networks.

To quantify the similarity with our responses we tested, to what extent the hits from Hsp90-responsive clusters are upregulated in these publicly available experiments. To this end we implemented a routine, which calculates a score (UpRegScore) on the scale from 100 (perfect up-regulation) to -100 (perfect down-regulation). It takes as input any list of genes, e.g. from a cluster to be tested, and determines to what extent each gene of this set is upregulated in a chosen microarray experiment. The UpRegScore is calculated based on the positions of these genes in a list of all genes ranked according to their differential expression with the most upregulated on top. It then compares the summed up positions for the investigated genes (AddedRealPositions) to the best possible (AddedIdealUpPositions) and worst possible scenarios (AddedIdealDownPositions), resulting in positive values up to +100 if the group is highly upregulated or negative scores if it is downregulated:

$$UpRegScore=100-200*\frac{AddedRealPositions-AddedIdealUpPositions}{AddedIdealDownPositions-AddedIdealUpPositions}$$

Statistical analysis was performed by generating 100 similar sized random gene lists, which were then evaluated with the same strategy to obtain the average value and the standard deviation. These values were then used to determine the significance level based on the calculated Z-score as described above. The values for 88 random genes are depicted in the Figures and other gene numbers led to very similar mean and standard deviations of the controls. Only for very small gene lists (below 20 genes) the standard deviation was getting larger, which impacts the significance of the UpRegScore for small clusters, such as *Daf-21*down_3 and *Daf-21*down_4.

### GO enrichment analysis

GO Enrichment Analysis was performed with the help of the webserver at http://geneontology.org/page/go-enrichment-analysis [39, 40]. Here we uploaded gene lists from individual clusters and the enrichment analysis was performed in the category “Biological Process” without the Bonferroni Correction. Results were ranked according to their p-values and the highest ranking terms, which showed significant enrichment (marked with “+” in the enrichment column and p-values<0.001), were included in the bar chart Figures with their respective enrichment factor as y-axis value.

### Microscopy of GFP-reporter strains

We obtained fluorescent reporter strains for some critical hit genes. As such we obtained the strains HZ455 (*sepa-1p*::SEPA-1::GFP + unc-76(+) [41]), RT688 (*pie-1p*::CAV-1::GFP + unc-119(+) [42]) and BC12422 (*zip-8p*::GFP + pCeh361 [43]) from the CGC, the strain SHK207 (*skr-5p*::SKR-5::GFP [44]) from Professor Sivan Henis-Korenblit (Bar-Ilan University, Israel) and the strains AU10 (*dod-24p*::GFP) and AU185 (*clec-60p*::GFP [37]) from Professor Frederick Ausubel (Harvard University). The strain MQD396, containing a *pud-1*.*1p*::GFP::PUD-1.1 reporter construct was obtained from Professor Dong Meng-Qiu (National Institute of Biological Sciences, Bejing). Non-integrated strains, like SHK207 and BC12422, were maintained by picking rollers or wildtype, respectively. With each strain we performed RNAi against Hsp90 and empty control and monitored differences in the fluorescence signal during the experiment at the indicated time. To visualize the differences, worms were mounted on 2% agarose pads and images were taken on a Zeiss Axiovert microscope equipped with a Hamamatsu camera (Hamamatsu Corporation).

### qRT-PCR for *skr-5* and *act-1*

Nematodes were harvested 2.5 days after placement of L1 larvae on RNAi plates. Isolation of total RNA from the nematodes was performed with the SV Total RNA Isolation System (Promega Corporation) following the manufacturer’s instructions. Primers used in the qRT-PCR experiments were designed with Primer-BLAST (https://www.ncbi.nlm.nih.gov/tools/primer-blast/). The final primer sequences were: *act-1*_fwd (AATCCAAGAGAGGTATCCTTA), *act-1*_rev (GATGGCGACATACATGGCT), *skr-5*_fwd (AATTGGTGCTGGCAGCCAC), *skr-5*_rev (GTTACCCAAGTTGAAAACGGCACG). qRT-PCR samples were prepared with the Brilliant III SYBR® Master Mix Kit (Agilent). The PCR program on the Agilent Mx3000p was selected as described in the user manual. After the PCR reaction, the amplified DNA was tested to confirm the purity and the correct length of the product. This was done by on-chip gel electrophoresis with the Agilent 2100 Bioanalyzer system. We used the expression values only in cases, where we could obtain a specific product at the right size. The experiments were performed in triplicates and averaged.

## Results

### Hsp90-downregulation leads to accumulation of chaperones and depletion of certain lectins

**Fig 1:**
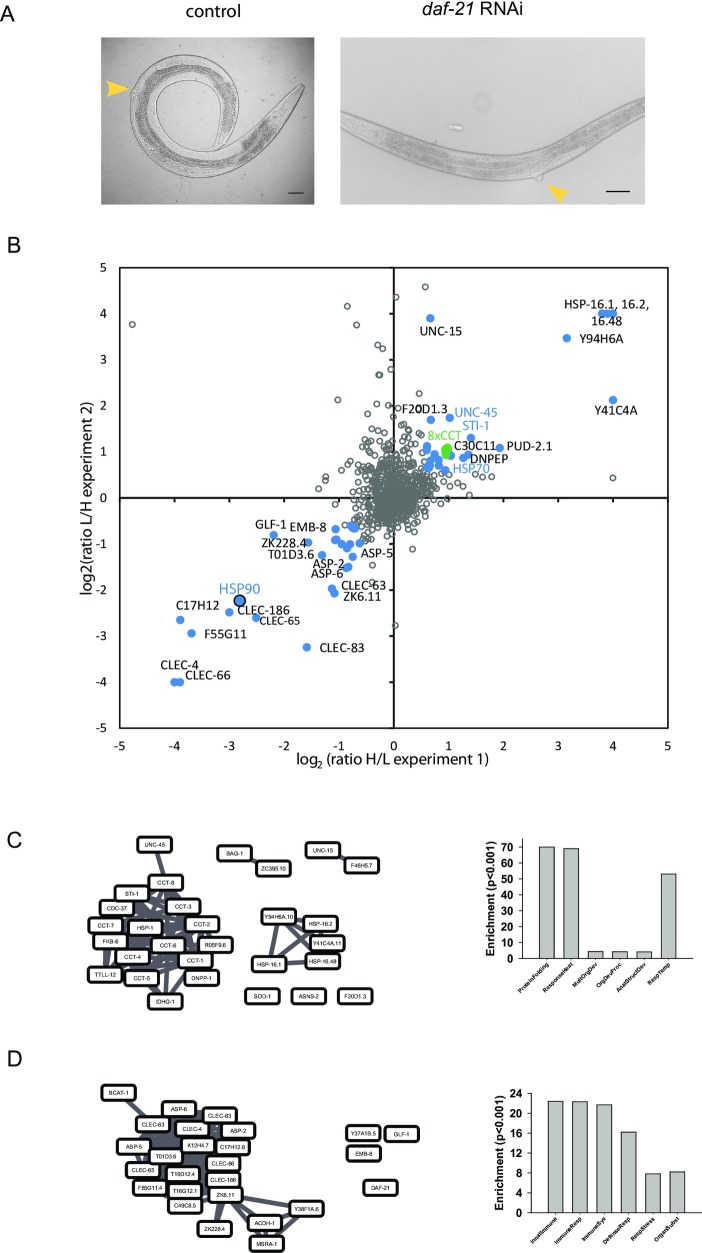
Influence of Hsp90-depletion on the *C*. *elegans* proteome. A) Control and Hsp90 RNAi treated N2 nematodes at the moment of harvesting for analysis. The developing phenotype can be seen at the vulva site (yellow arrow) and the Hsp90-depleted nematodes are slightly smaller at that stage. B) Proteomic changes based on isotope-labelled mass spectrometry. The two independent experiments are plotted in the two dimensions. Blue spots indicate genes, for which the identification scores based on the number of detected peptides imply a reliable quantification. C) Network of upregulated proteins and corresponding GO enrichment analysis. The network was generated based on hit-to-hit relationships in a coexpression database as described in the Materials and Methods section. D) Network of downregulated proteins and corresponding GO enrichment analysis. The network was constructed with identical settings as in C.

We first used mass spectrometry to investigate changes in the nematode’s proteome upon Hsp90-depletion. To perform a mass spectrometric comparison between Hsp90-depleted and control nematodes, the *daf-21* dsRNA-expressing bacterial strain was grown on plates containing solely ^15^NH_4_SO_4_ as nitrogen source. The control population was grown on NGM-plates containing ^14^NH_4_SO_4_. RNAi against Hsp90 leads to slower development and sterility. Nematodes were harvested when they approached L4 larval stage with Hsp90-depleted nematodes being slightly smaller at the moment of harvesting (Fig 1A). After washing the nematodes off the respective plates the worms were lysed and equal amounts of the two isotope-specific samples were combined. The samples were then subjected to mass spectrometric analysis performing a direct comparison of the identified peptides in a single mass spectrometry experiment. To remove any impact the nitrogen source may have, the experiment was then repeated with exchanged nitrogen sources (^15^NH_4_SO_4_ for control, ^14^NH_4_SO_4_ for Hsp90-depletion) so that altogether two datasets were obtained to describe this system.

About 400 proteins could be quantified and we compared their relative levels. Proteins upregulated after Hsp90-depletion in both experiments included many known heat-shock proteins and other proteins associated with the chaperone system (Fig 1B, S1 Table). Similar to previous reports, these proteins were the strongest upregulated group, indicating that the induction of the heat-shock response is a significant event after depletion of Hsp90 [8]. As such, the small heat-shock proteins HSP-16.2, HSP-16.1 and HSP-16.48 are highly overexpressed (log_2_ of about 4). STI-1, UNC-45 and other cochaperons of Hsp90 also showed increased protein levels, as does the protein folding machine CCT/TriC (log_2_ of about 1, Fig 1B). Additional proteins found to be enriched in Hsp90-depleted nematodes were UNC-15, Y94H6A.10, Y41C4A.11 and F20D1.3. Interestingly, most of these differentially expressed proteins, including Y94H6A.10 and Y41C4A.11, can be clustered into a network using information from a transcriptional coexpression database (Fig 1C, left side). This implies that these proteins are commonly found to be regulated in the same direction. Testing the upregulated proteins for enrichment of GO-Terms also shows a strong enrichment in “protein folding” and “unfolded protein binding” (Fig 1C, right side), as expected from the nature of the included proteins.

Several proteins also showed reduced levels in Hsp90-depleted nematodes (Fig 1B, S2 Table). This group included several lectin-like genes (such as CLEC-4, CLEC-65, CLEC-66, CLEC-83) and aspartate proteases (such as ASP-2, ASP-5 and ASP-6). Successful knock-down was confirmed by Hsp90/DAF-21 showing up in this group as well (log_2_ of -2.8). Here most proteins can be clustered based on their known coexpression (Fig 1D, left side) and GO enrichment analysis indicates that these proteins are usually coregulated as part of the innate immune response (Fig 1D, right side) [38, 45–48]. This is true for the C-type lectins and aspartate proteases. Furthermore many of the unassigned proteins in this group, such as K12H4.7, T19D12.4, F55G11.4, C49C8.5, ZK6.11 and ZK228.4 also are associated with the innate immune response according to Wormbase [49]. Thus *C*. *elegans* obviously responds to Hsp90 RNAi with specific proteins originating from defined transcriptional clusters related to the heat-shock response and the innate immune response.

### Gene expression changes upon depletion of Hsp90 highlight the impact on developmental processes

To better define the connection between these clusters, we investigated the full transcriptional program under this condition by employing Affymetrix DNA microarrays. Three experiments were performed under conditions similar to the proteome analysis by harvesting the nematodes shortly before they reached fertility. We averaged the expression differences for the biological replicates and obtained the genes that were most strongly up- or downregulated from the 26,959 sampled transcripts (Table 1). To calculate transcriptional networks, we used the top 250 genes in each direction and clustered these genes with the help of public coexpression databases as described in Materials and Methods [26]. To test the significance of the obtained networks, we compared their connectivity with 100 networks of random genes. Indeed, in both Hsp90-dependent networks, the connectivity parameters, such as the percentage of hits included (S1A Fig.) and the overall number of connections (S1B Fig) are much higher than for random genes (748 and 952 connections versus 12 and 16, p<0.001). This shows that the hits of this response appear commonly coexpressed. We further tested whether up- or downregulated genes outside the 250 hits can be successfully predicted from the coexpression database, a feature which shows the completeness of the transcriptional response. We quantified this ability with a score between -100 (worst possible prediction for hit genes 250–300) and 100 (perfect prediction for hit genes 250–300). The determined score is higher than 90 for both directions, while being close to 0 for the similar sized random gene groups (S1C Fig), showing that almost all predicted genes behaved as expected (p-value < 0.001). This confirms that both directions of our microarray experiment contain genes that are commonly coexpressed based on the used coexpression database [28]. This is not only true for the averaged hit lists but also for each of the biological replicates (S1C Fig), which confirms the high data quality of each individual experiment. The predictive ability evident from the high CoRegScores can only be explained if the Hsp90-depletion induced response shares many similarities with the hundreds of microarray experiments which were used to calculate the coexpression correlations in the modSEEK database [28].

**Table 1:** Averaged expression differences of strongly affected genes after *daf-21* RNAi. The genes depicted are the most strongly affected genes. The three experiments were averaged to obtain the average expression change and the standard deviation. These genes are part of the network structures of Fig 2. The weaker hits are truncated in this table, but included in the networks in Fig 2. The assignment to the clusters is based on the cluster description in Fig 2. “0” implies that the gene is outside of all marked clusters, “+” connections imply that the gene is in-between marked clusters and “–” implies that this particular gene was not found in the used coexpression database. In the column “strain” all currently available fluorescent reporter strains for these hits are shown, but strains in brackets were not used in our study.

Upregulated Gen	Transgene Strain	Average	STD	*Daf-21*Up	Downregulated Gen	Transgene Strain	Average	STD	*Daf-21*Down
arf-1.1	-	6.01837	0.978103	1	tbh-1	(MT9971)	-2.56878	2.57384	1
Y41C4A.11	-	5.68503	0.463387	1	Y57G11B.5	-	-2.49476	2.46702	1
Y47H10A.5	-	4.5152	1.60171	1	clec-223	-	-2.4418	2.47944	1
C50F7.5	(SD1583)	4.19376	1.28643	1	F59D8.3	-	-2.41413	1.70888	-
F15B9.6	-	3.95603	1.10137	1	ZK813.3	-	-2.3442	2.07643	1
skr-5	SHK207	3.8018	1.12279	1	F49E12.1	-	-2.33443	2.44208	-
Y37H2A.14	-	3.76196	0.726931	0	clec-209	-	-2.2619	1.69682	4
sdz-35	-	3.59986	0.951894	1	W04A4.2	-	-2.25409	2.23416	1
clec-60	AU185	3.53321	1.9834	1	cyp-13A6	-	-2.24297	3.3709	0
T26H5.9	-	3.51657	1.14567	-	C39D10.7	-	-2.23089	2.65949	1
fbxa-163	-	3.45604	1.59722	1	cav-1	RT688	-2.21036	1.0788	2
R03H10.6	-	3.35083	0.623473	1	vit-4	-	-2.20557	2.55327	3
K08D10.10	-	3.30692	0.91793	-	Y62H9A.6	-	-2.16989	2.44449	1
ZK355.8	-	3.23513	2.23024	-	dod-24	AU10	-2.1679	1.88533	4
clec-61	-	3.19526	1.54801	1	C25A8.4	-	-2.10898	2.4159	-
F22E5.6	-	3.08391	0.635536	1	K10C2.8	-	-2.07235	1.45343	-
B0563.9	-	3.07138	2.39947	-	T04G9.7	-	-2.06123	2.48285	1
Y68A4A.13	-	2.99054	2.03498	-	Y62H9A.4	-	-2.05896	2.06525	1
col-176	-	2.94957	2.66572	2	sepa-1	HZ455	-2.05658	1.49506	2
R07C12.1	-	2.93562	0.611104	1	C35E7.5	-	-2.0464	1.21709	2
C08E8.4	-	2.79468	0.755811	1	skr-7	-	-2.04313	1.10403	1+2
F29G9.14	-	2.78478	0.923283	-	pes-2.1	-	-2.03482	1.36011	2
C08E3.1	-	2.78009	1.5147	0	sri-40	-	-2.02298	1.54135	1
C17F4.12	-	2.75546	1.97348	2	D1054.11	(SD1981)	-2.01782	2.16539	1
dct-3	-	2.71538	0.490552	-	C44B7.5	-	-2.01272	2.04742	1
bli-1	(CH1445)	2.69926	2.15158	2	ZC373.2	-	-2.0072	2.26472	1
sqt-1	-	2.68336	2.36365	2	efn-3	-	-2.00689	1.79793	1
col-73	-	2.68196	1.31729	0	ets-7	-	-1.99975	1.08082	2
tts-1	-	2.62731	1.48224	1	Y48E1B.8	-	-1.99174	1.2993	0+1
clec-180	-	2.58966	2.50659	2	T27A10.8	-	-1.98758	1.45337	-
dao-2	-	2.57777	2.30527	2	F48E3.4	-	-1.98397	2.74176	1
pqn-63	-	2.56242	1.92395	2	ZK813.2	-	-1.94439	1.6098	1
dpy-13	-	2.55089	1.87921	2	vet-6	-	-1.94433	1.06462	2
Y105C5A.12	-	2.54054	1.38919	1	Y39G10AL.1	-	-1.94291	0.778773	2
F13D11.4	-	2.53557	1.98456	2	sdz-25	-	-1.94233	1.38382	2
col-38	-	2.53333	1.71205	2	vit-3	-	-1.9359	2.34383	1
B0024.4	-	2.53134	1.13112	1	D1086.6	-	-1.93088	1.66361	-
ugt-18	-	2.49466	0.887937	1	F55B11.3	(SRS86)	-1.92928	2.06282	1
tag-297	-	2.49185	1.78713	2	ZK813.7	-	-1.92374	1.98332	1
F33H12.7	-	2.48457	1.03716	-	Y62H9A.5	-	-1.9195	2.61316	1
F44E2.4	-	2.47906	2.21763	2	B0513.4	-	-1.88055	2.34603	1
F48G7.8	-	2.47772	1.01412	2	K11D12.13	-	-1.87013	1.85672	-
col-138	-	2.46287	1.70462	2	vit-1	-	-1.8571	2.33645	1
lpr-3	-	2.46111	2.68673	2	nas-20	-	-1.8557	1.43733	1
mlt-11	-	2.43061	2.54662	2	K07A1.6	-	-1.84355	1.80729	1
B0284.1	(BC15640)	2.42306	0.804259	0	F57C2.4	-	-1.83249	1.67229	1
Y94H6A.10	-	2.41241	1.07078	1	D1054.10	-	-1.81174	1.8609	1
R02F11.1	-	2.41111	2.02839	2	acdh-2	-	-1.79298	2.37892	0+1
col-175	-	2.40976	1.29287	2	F15E11.15	-	-1.77703	2.61117	-
tsp-1	-	2.39753	1.1672	1	C17G1.2	-	-1.77345	2.15736	1
T28H10.3	(RW10819)	2.39438	1.05975	1	ttr-42	-	-1.7662	1.31818	-
M60.7	-	2.39325	1.40075	1	clec-266	-	-1.75624	1.65512	2
F10A3.1	(BC13458)	2.38407	0.650951	1+2	linc-121	-	-1.75054	1.21151	-
rol-1	-	2.38016	1.67645	2	sdz-28	(RW10371)	-1.74133	0.828096	2
E01G4.6	-	2.37237	2.05367	2	C04B4.2	(BC15374)	-1.73929	1.57356	2
acn-1	-	2.36373	1.87353	2	skr-10	-	-1.73509	1.33333	2
ptr-4	-	2.35218	2.04175	2	D1086.11	-	-1.71749	1.85678	-
Y65B4BL.1	-	2.33269	2.28006	2	F54F7.2	-	-1.71409	1.33282	1
H42K12.3	-	2.331	2.50187	2	clec-218	-	-1.71321	0.870155	3
col-169	-	2.33062	3.05638	-	F02E8.4	-	-1.71192	1.65181	2
hsp-70	-	2.33044	1.01536	1	T12B5.15	-	-1.70195	0.941428	-
C54D10.14	-	2.32684	0.40607	1	R04D3.3	-	-1.67453	1.71792	1+2
col-49	-	2.31983	1.81372	2	Y62H9A.3	-	-1.66364	1.89092	1
B0462.5	-	2.31955	1.40587	-	pud-1.1	MQD396	-1.65744	2.70827	-
ZK84.1	-	2.31004	2.09149	2	cdc-25.3	(BC12401)	-1.65662	1.28574	1+2
Y34F4.4	-	2.29565	1.02139	1	F55B11.2	-	-1.6509	1.4361	1
col-65	-	2.29107	1.22273	2	col-135	-	-1.63889	1.0186	-
scl-2	-	2.27978	1.3477	1	R03G8.6	-	-1.631	1.34914	1
abu-14	-	2.27971	1.84813	2	flh-3	-	-1.6308	1.60568	2
T01B7.13	-	2.27827	1.93391	-	F13G11.3	(SD1973)	-1.62962	1.36361	-
bli-2	-	2.27603	2.26035	2	ent-5	-	-1.62676	1.54476	1+2
ZC239.14	-	2.27466	1.25068	1	C49F5.6	-	-1.62265	1.44651	2

**Fig 2:**
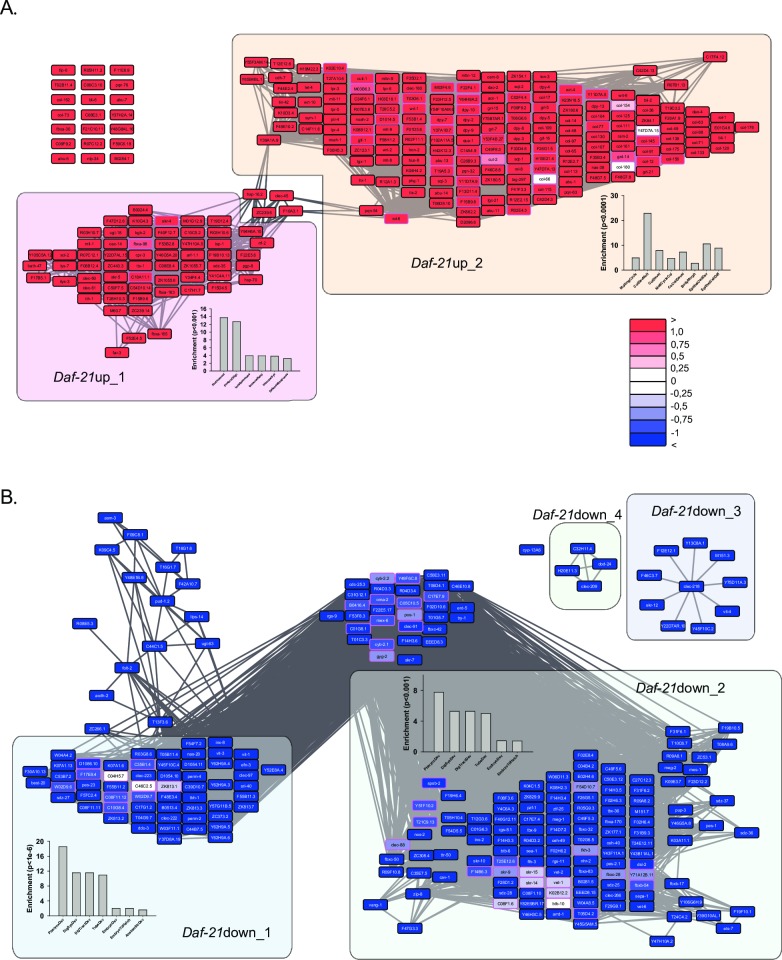
Transcriptional networks affected by RNAi against *daf-21*. Networks for upregulated (A) and downregulated (B) genes after Hsp90-depletion. The networks were constructed as described in the Materials and Methods section. The color code uses four shadings of blue and red to indicate the expression differences with darkest blue being log_2_(DiffExp) < -1, lightest blue being log_2_(DiffExp) < -0.25, darkest red being log_2_(DiffExp) > 1 and lightest red being log_2_(DiffExp) > 0.25. In between 0.25 and -0.25 the nodes are white as indicated in the legend. Large clusters have been subjected to GO-term enrichment analysis and the results are depicted adjacent to the respective cluster in the network figure.

**Fig 3:**
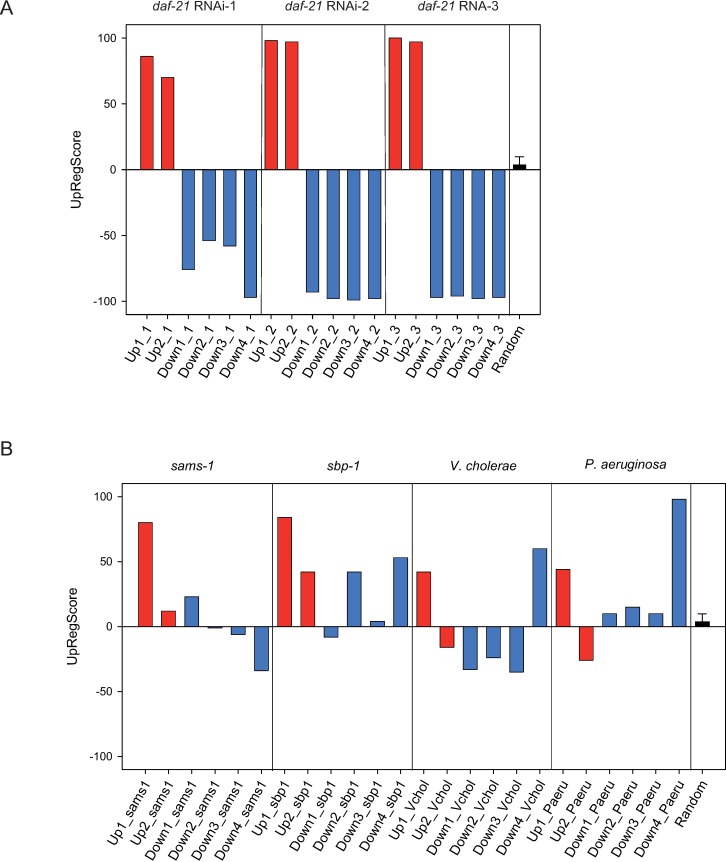
Regulation of separated clusters during *daf*-21 RNAi-response and other conditions. A) Comparison between the three replicates regarding the up- or downregulation of specific clusters. The included genes are used to determine the UpRegScore of the respective cluster in each of the experiments as described in Materials and Methods. The score was calculyted as described in the Materials and Methods section. The results for the respective replicate on the single-gene basis are show in S2 and S3 Figs and S4B Fig) Calculation of the UpRegScore for each gene cluster in the microarray experiments for RNAi against *sams-1* or *sbp-1* (left side), *V*. *cholerae* VC109/VC110 exposure and *P*. *aeruginosa* exposure (right side). The corresponding results on the single-gene basis are shown in S5–S8 Fig.

We then visualized the connected hits as clustered networks (Fig 2A and 2B). The genes upregulated after Hsp90-RNAi arrange in two clusters (*Daf-21*up_1 and *Daf-21*up_2), which are minimally connected by coexpression relationships (Fig 2A). One of the clusters (*Daf-21*up_1) connects the genes of the heat-shock response (*hsp-16*.*2* and *hsp-70*) previously shown to be induced after RNAi against Hsp90 [5, 8, 15] with 61 other genes. According to GO-term enrichment analysis and confirmed by individual searches, this cluster contains many genes related to the innate immune response (Fig 2A). It also includes the most strongly upregulated genes *arf-1*.*1* (60-fold), Y41C4A.11 (50-fold), Y47H10A.5 (32-fold) and C50F7.5 (8-fold) (Table 1). The large network of *Daf-21*up_2 instead originates from the slower development of the Hsp90-depleted nematodes. It contains genes involved in larval development and collagen and cuticle formation. Based on GO-term enrichment the genes from this cluster are mostly related to the molding cycle, cuticle development, and also to the development of individual tissues like pharynx and intestine (Fig 2A). We then tested whether these two clusters are upregulated in all three replicates alike. Indeed, in all replicates, the innate immune/heat-shock response cluster *Daf-21*up_1 is strongly upregulated (UpRegScores of 86, 98 and 100). While the upregulation of *Daf-21*up_2 is slightly weaker in the first experiment (UpRegScore of 70, 97 and 97, respectively), it is still clearly significant when compared to 100 random gene sets (UpRegScore: 3.7 ± 6.2, p<0.001) (Fig 3A). Thus the three biological replicates generate similar responses on the clustered level and this correlation is also evident from the same top hits being upregulated in all three experiments (S2–S4 Figs). Nevertheless, the biological replicates differ in the strength of their transcriptional responses, which may be caused by a variable RNAi-induced knock-down among replicates or slight differences of the developmental state at the harvesting moment.

### Downregulated genes are involved in germline development and innate immune response.

We also visualized the clusters of genes downregulated after Hsp90-depletion (Fig 2B). Here two main clusters can be separated (*Daf-21*down_1 and *Daf-21*down_2) based on the database-retrieved coexpression relationships. Contrary to the upregulated dataset the connections between the two main clusters are much stronger. This implies that they may not be independently regulated. Two very small clusters (*Daf-21*down_3 and *Daf-21*down_4) are formed separately. Only the gene *cyp-13A6* is not connected to any partner gene within the clustered network (Fig 2B). Also for this side of the response, the three replicates show striking similarities and the UpRegScores for all clusters are between -54 and -99 compared to 3.7 ± 6 for random genes (p<0.001). Again the first replicate is showing a weaker response. According to GO-enrichment analysis and based on single-gene queries, the two main clusters contain genes that are involved in the development of different tissues from pharynx and epithelial cells to embryo development (*Daf-21*down_1 and *Daf-21*down_2). This also is evident from Wormbase information, where many genes in these clusters are associated with processes in the germ line (C39D10.7, Y62H9A.4, Y62H9A.6, ZC373.2 and C17G1.2), the spermatheca (clec-223, ZK813.3 and F55B11.3), or in the early embryo prior to gastrulation (pes-2.1, T04G9.7, vet-6 and F55B11.2). Thus the main part of the lower expressed transcriptional response reflects the slower developmental progress of the Hsp90 RNAi-treated nematodes and the stalled development of its gonads and embryos. In the first replicate where the emphasis of the response is shifted towards the immune response, highly inducible genes are primarily seen in the *Daf-21*down_4 cluster. Some of these genes, like the gene *dod-24*, have been described as DAF-16-regulated genes related to the innate immune response [50] and responsive to stress conditions [51]. Likewise, the *dod-24* connected genes H20E11.3 and C32H11.4 are part of innate immune responses [47] along with the gene *clec-209*, which was found as top-ranking gene in innate immune responses against *Vibrio cholerae* [36]. Thus in the downregulated clusters, genes that are related to the innate immune response are set apart from the many genes that are connected due to their coexpression during the nematode’s developmental process.

### Expression changes after Hsp90-depletion overlap with described immune responses.

We next aimed at identifying conditions where similar expression patterns had been observed. We realized that several of the genes most prominent in our study had also been prominent in other microarray experiments, including a very recent study on the innate immune response [52]. We thus compared our response to these microarray experiments which were performed to study the effects of a depletion of SAMS-1 and SBP-1 [33, 52]. We analyzed the publicly available data for RNAi against *sams-1* and tested the similarity to Hsp90-depletion by plotting the *sams-1*-related expression values onto the networks obtained from our microarray data. To quantify the overlap in the respective clusters, we calculated the UpRegScore for each of our clusters (Fig 3B, S5 Fig). Clearly the cluster *Daf-21*up_1 is regulated similarly in these two experiments (UpRegScore of 80, p<0.001) while the other clusters do not show an overlapping response (S5 Fig). The same is true for the response to depletion of *sbp-1* (UpRegScore of 84, p< 0.001, Fig 3B, S6 Fig). Thus Hsp90-depletion induces significant parts of the innate immune response in a manner similar to other genes previously connected to the normal regulation of this response, like *sams-1* and *sbp-1* [33]. Furthermore, the exclusive induction of cluster *Daf-21*up_1 in the *sams-1* and *sbp-1* RNAi experiments confirms that *Daf-21*up_1 and *Daf-21*up_2 are indeed regulated entirely independent from each other. To directly test the overlap of *Daf-21*up_1 with the pathogen-induced immune response, we evaluated the response of our clusters during immune responses against *Vibrio cholerae* and *Pseudomonas aeruginosa* [36, 37] (Fig 3B, S7 and S8 Figs). Indeed many of the genes of cluster *Daf-21*up_1 are also induced in response to these pathogenic bacteria (UpRegScore of 41, p<0.001 for *V*.*cholerae* and UpRegScore of 44, p<0.001 for *P*. *aerginosa*) but the overlap in this cluster is not as high as in the *sams-1* and *sbp-1* RNAi-induced responses. Again no significant similarity can be observed in the other clusters. Thus one part of the differentially expressed genes clearly shows that RNAi against Hsp90 strongly induces the innate immune response. The second set of differentially regulated genes instead shows the changes to the development of the nematode and also the impaired development of its embryos in an independently regulated response.

### Hsp90-depletion induced changes to the immune response are evident in intestinal tissues

**Fig 4:**
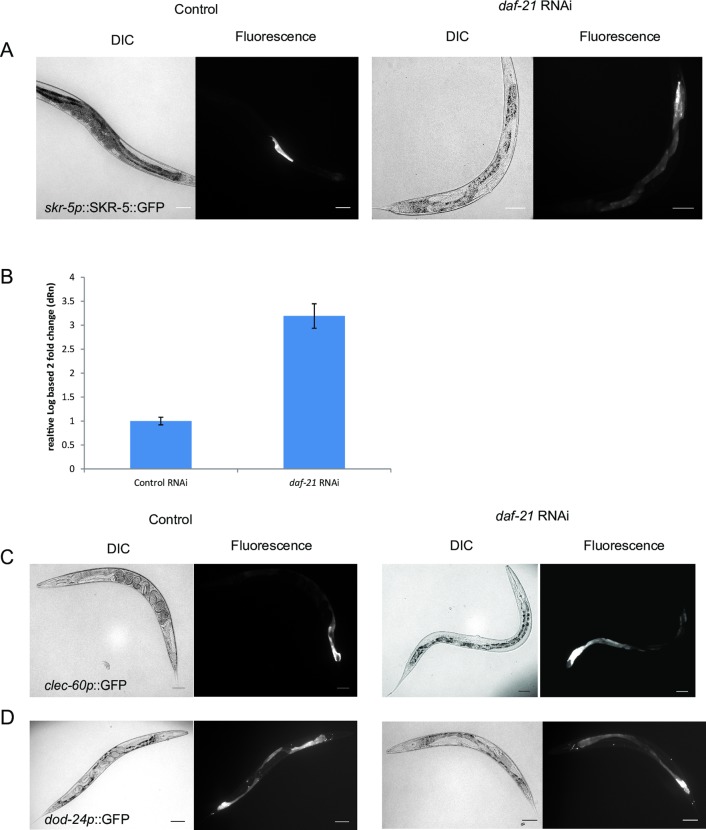
Immune response genes respond to *daf-21*-RNAi in intestinal tissues. DIC and fluorescence images of Hsp90-treated (right panels) and control nematodes (left panels) are shown. A) SHK207 nematodes were imaged to visualize SKR-5::GFP protein. Control nematodes showed rare expression in Int5, Int6 and Int7 cells. Hsp90-depleted nematodes generally showed induction in the full intestine. B) qRT-PCR has been performed for *skr-5* in control nematodes and in Hsp90-depleted nematodes as described in Materials and Methods. C) AU185 nematodes were imaged to visualize *clec-60p*-driven GFP expression. Control nematodes express in Int8 and Int9 cells and rarely in more anterior intestinal cells (left panel). Hsp90-depleted nematodes show induction in most parts of the intestine (right panel). D) AU10 nematodes were imaged to visualize expression of *dod-24p*-driven GFP. Control nematodes show induction in all parts of the intestine (left panel). Expression is substantially reduced in Hsp90-depleted nematodes (right panel). The scale bar represents 100 μm.

Having identified genes which are differentially affected after RNAi against Hsp90, we aimed at testing some of the main hits in fluorescent reporter strains. This also helps to define the tissues in which Hsp90-related changes can occur. Previous experiments had successfully visualized the induction of the heat-shock response in body wall muscle cells and intestinal cells upon Hsp90 knock-down [5, 8]. For this aim, we initially obtained publicly available reporter strains for genes that are prominent in our immune response clusters. We aimed at investigating the strongest affected genes and preferred translational GFP-fusions over transcriptional reporters. The first strain contains a GFP-fused version of SKR-5. SKR-5 is a Skp1-homolog protein [53] expressed in intestinal tissues and upregulated 6–30 fold (log_2_: 3.8 ± 1.1) in our microarray experiments. We subjected these nematodes to RNAi against Hsp90 and control RNAi and then compared the fluorescence intensity and localization to see whether differential regulation can be observed in response to Hsp90-depletion. We see GFP-SKR-5-expression in some control treated SHK207 nematodes during later larval stages in posterior parts of the intestine as previously reported [44]. The Hsp90-depleted SHK207 nematodes instead show much stronger fluorescence in the entire intestinal tissue with most nematodes now being fluorescent in all cells of the intestinal tube (Fig 4A). The upregulation of this gene also is evident in qRT-PCR assays, where a three-fold upregulation can be observed (Fig 4B). We next tested a transcriptional *clec-60* reporter strain. CLEC-60 is a C-type lectin known to be regulated as part of the innate immune response. It is expressed in larval intestine and in adults in the Int8 and Int9 cells [54, 55]. In control RNAi experiments, we observed the reported behavior. Upon RNAi against Hsp90, the fluorescence is strongly increased (Fig 4C). To exclude differences that may arise from the slow growth of Hsp90-RNAi treated nematodes, we tested if earlier larval stages of the control-treated nematodes show an expression with similar intensity to that of Hsp90-dsRNA treated nematodes. However, this was not the case for these reporter strains (data not shown), implying that the observed upregulation in intestinal tissues reflects the consequences of the Hsp90-depletion. We then tested a transcriptional reporter strain containing *dod-24p*::GFP. DOD-24 is an epoxide hydrolase connected to the innate immune response [51]. This strain shows high expression throughout the whole intestine under normal growth conditions (Fig 4D, left side). After treatment with Hsp90-RNAi, the expression in the Int3 to Int7 intestinal cells is strongly reduced while it is retained to a weaker extent in the very anterior and posterior intestinal cells (Fig 4D, right side). Thus, in these three reporter strains which change their expression as part of the nematode’s immune response, the intestinal expression of fluorescent markers correlates well with the expression analysis, confirming the influence of Hsp90-RNAi on this immune response pathway.

### Genes from developmental clusters are affected in the nematode’s gonads and embryos.

**Fig 5:**
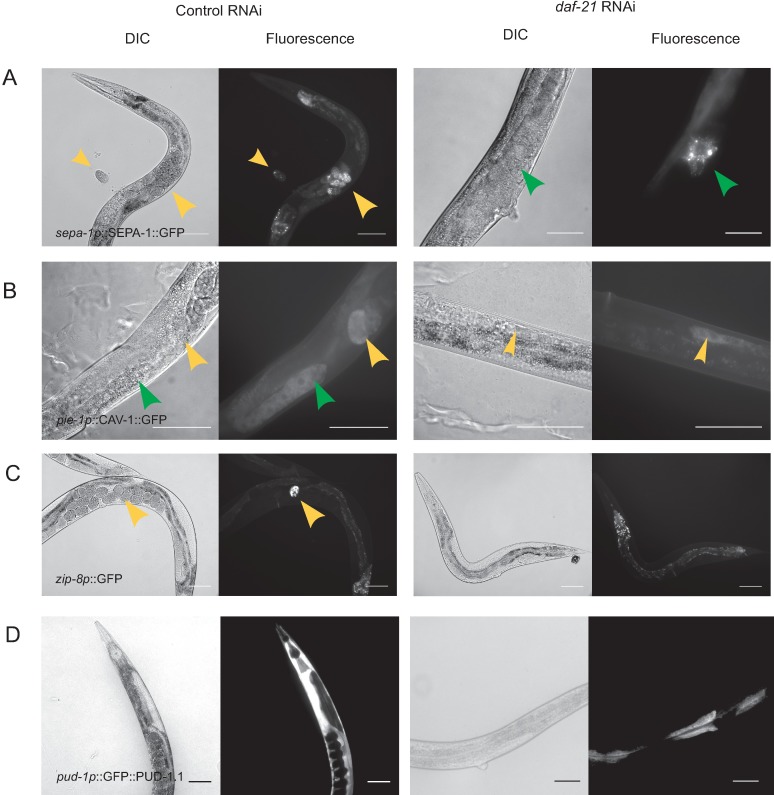
Genes from cluster *daf-21*Down_2 are suppressed by Hsp90 RNAi in gonads and embryos. A) HZ455 nematodes were imaged to visualize the expression and subcellular localization of SEPA-1 protein. Yellow arrows indicate the position of developed embryos, while green arrows indicate the fluorescence prior to passage through the spermathecum. B) RT688 nematodes were imaged to visualize subcellular localization of CAV-1 protein. The CAV-1 protein in the meiotic zone of the gonad arm is indicated in both panels with a green arrow, while yellow arrows indicate the position of developed embryos. C) BC12422 nematodes were imaged to visualize the cells expressing *zip-8*. The scale bar represents 100 μm. Yellow arrows show the position of a *bzip-8*::GFP expressing embryo. D) GFP::PUD-1.1 can be observed in intestinal tissues in later larval stages. After Hsp90 RNAi this expression is strongly diminished. The scale bar represents 100 μm.

We then tested strains for hits that cluster in the early developmental networks. Here we initially picked a translational reporter strain for SEPA-1, a protein, which mediates autophagy of P-granules in early embryos [41, 56]. The expression of this gene product, which is degraded once embryos reach 100 cells, is decreased in our microarray data upon Hsp90 knock-down. Specifically SEPA-1 (Fig 5A) can be observed in the syncytial gonad arms and in the embryos prior to and just after passage through the spermathecum. We tested the influence of Hsp90-depletion on the localization of this protein and its expression. Indeed, we see reduced fluorescence of this stage-specific protein and incomplete development of gonad arms containing this protein. Moreover, the embryos did not show SEPA-1 containing structures after passage through the spermathecum, implying that fertilized oocytes are not generated correctly. We then tested a translational reporter for the caveolin homolog CAV-1 [42] from the *Daf-21*down_2 cluster. This protein is required during the first cell division and then quickly disappears from the embryo. Oocytes approaching the spermatheca in Hsp90-depleted worms contain CAV-1, but are reduced in size and no fertilized oocytes expressing CAV-1 can be seen after the passage through the spermatheca (Fig 5B). However, in few cases, very brightly fluorescent oocytes are found containing high amounts of CAV-1 without evident structural organization (data not shown). These likely represent the previously observed endomitotic oocytes [4]. These changes show that Hsp90 depletion significantly impacts the developing oocyte prior to fertilization. We finally tested the expression of the bZIP-like transcription factor ZIP-8 [43], which is also present in the *Daf-21*down_2 cluster. Based on the fluorescent reporter strain BC12422, p*zip-8* is activated in early embryos and is also present at later stages in the adult intestine. We did not observe p*zip-8*::GFP expression in oocytes of Hsp90-depleted nematodes, while it was clearly visible in L4440-control treated nematodes (Fig 5C). Thus the reduced expression levels within this cluster point to the failure of Hsp90-depleted nematodes to develop functional oocytes.

We finally tested a reporter strain of the downregulated gene *pud-1*.*1*. While *pud-1*.*1* not included in the main clusters of the downregulated network, it is loosely connected to cluster *Daf-21*down_1. PUD-1.1 is one of two identical PUD-1 proteins of unknown function that are known to be differentially regulated during aging and development [57]. In control nematodes, GFP-PUD-1.1 is found in the intestine at later larval stages [58]. We see a decrease of fluorescence in the intestine of Hsp90 RNAi-treated nematodes, implying that the upregulation in developing adults does not occur in Hsp90-depleted nematodes (Fig 5D).

These strains collectively confirm that processes during the development of oocytes are affected by depletion of Hsp90. This apparently happens in addition to the changes in intestinal tissues, where heat-shock response and innate immune response are observed.

## Discussion

Depletion of Hsp90 by RNA interference leads to various morphological and transcriptional changes in *C*. *elegans*. The combined phenotype of the Hsp90 knock-down includes developmental changes to gonad and vulval structures, induction of the heat-shock response, changes to muscle ultrastructure, and, as observed here, the induction of the innate immune response. Based on the function of Hsp90 as a regulator of signaling kinases and transcription factors, these diverse changes could be caused by multiple Hsp90-dependent processes. Here we used whole genome expression data to define pathways that are linked to Hsp90-depletion.

### Separate transcriptional pathways are induced by RNAi against Hsp90

To understand the induction of separate pathways, it is important to organize the differentially expressed genes into their respective expression clusters and then attempt guesses on transcription factors for the coexpressed gene groups. We recently implemented a gene clustering approach that was based on the use of genome-wide coexpression data. This yielded information-rich networks in yeast that clearly separated independent clusters and thus divided hit lists into separate transcriptional units [26, 27]. Potential transcription factors could then be derived from lists of target genes as available from YEASTRACT [26, 59]. Here we apply the same clustering strategy to the multicellular nematode *C*. *elegans* and like in yeast information-rich networks are obtained with all tested parameters proving a highly significant network and cluster formation. In this study, the *C*. *elegans* response to Hsp90 knock-down can be separated into multiple distinct clusters that can be further defined as independent based on the very low interconnection-numbers. This is supported by independent microarray experiments, which trigger only part of the Hsp90-depletion response. GO-term enrichment and individual searches further show that this clustering approach also separates the genes according to distinct functional processes. The reporter strains used in this study validate our organization strategy since genes from the same cluster behave similar regarding their tissue-specific expression and their fluorescence changes after Hsp90 RNAi. By this approach the response to Hsp90-depletion can be separated into one part that reflects a strongly induced innate immune response and into another part that reflects the slower and incomplete development of the nematodes and their reproductive structures.

### HSPs are upregulated on the proteome and transcriptome level

The induction of the heat shock response after Hsp90-depletion has been observed in reporter strains before [5, 8] and our study confirms this on the protein and mRNA levels. The transcriptional networks overlap with the proteomic response in particular for the heat-shock proteins HSP-16.1, HSP-16.2 and HSP-70. On the protein level the wider chaperone network is elevated, including many of the Hsp90 cofactors such as CDC-37, UNC-45, STI-1, ZC395.10 and FKB-6. Even though *hsp-16*.*2* and *hsp-70* are included in the transcriptional networks, the expression changes for most chaperones are barely evident on the mRNA level. This also is true for Hsp90 itself, whose mRNA is only weakly suppressed at the moment of harvesting (log_2_(DiffExp) = -0.15). This may imply that the chaperone system in the harvested nematodes has already built up a new balance at the beginning of phenotype-development. The heat-shock response certainly is not the strongest response on the transcriptional level at the moment of harvesting as other transcriptional clusters are more prominent among the top 250 genes.

### Hsp90-depletion induces the innate immune response in intestinal cells

The induction of the immune response is much more evident at the harvesting stage. Here, the upregulation of some genes, such as Y41C4A.11 (log_2_ of 5.69) and Y94H6A.10 (log_2_ of 2.42) from cluster *daf-21*Up_1 (innate immune response/heat-shock response) can also be observed on the proteomic level (log_2_ of 3.06 and log_2_ of 3.31, respectively). While Y41C4A.11 is a distant *C*. *elegans* homolog of the lipopolysaccharide-induced TNF-factor alpha, the function of Y94H6A.10 is unknown but its general coexpression with the heat-shock response and the innate immune response is evident in all experiments of this study. In microarrays and mass spectrometry approaches applied in this study, immune response genes are found to be both upregulated and downregulated. As such, several C-type lectins of the innate immune response are reduced after RNAi-treatment against Hsp90 [38, 45–48]. In general, most of the proteins found at reduced levels in our mass spectrometry experiments share a functional or coexpression connection to components of the innate immune response. This result holds true at the transcriptomic level, as many upregulated genes from cluster *daf-21*Up_1 (C08E8.4, Y47H10A.5, B0024.4, F15B9.6) can also be found upregulated in other cases where the innate immune response is activated. This is similar to the immune response after contact with *Vibrio cholerae* [36] or *Pseudomonas aeruginosa* [37] and for the response to RNAi against *sams-1* and *sbp-1* [52], two genes that are apparently able to suppress the innate immune response like *daf-21*.

**Fig 6:**
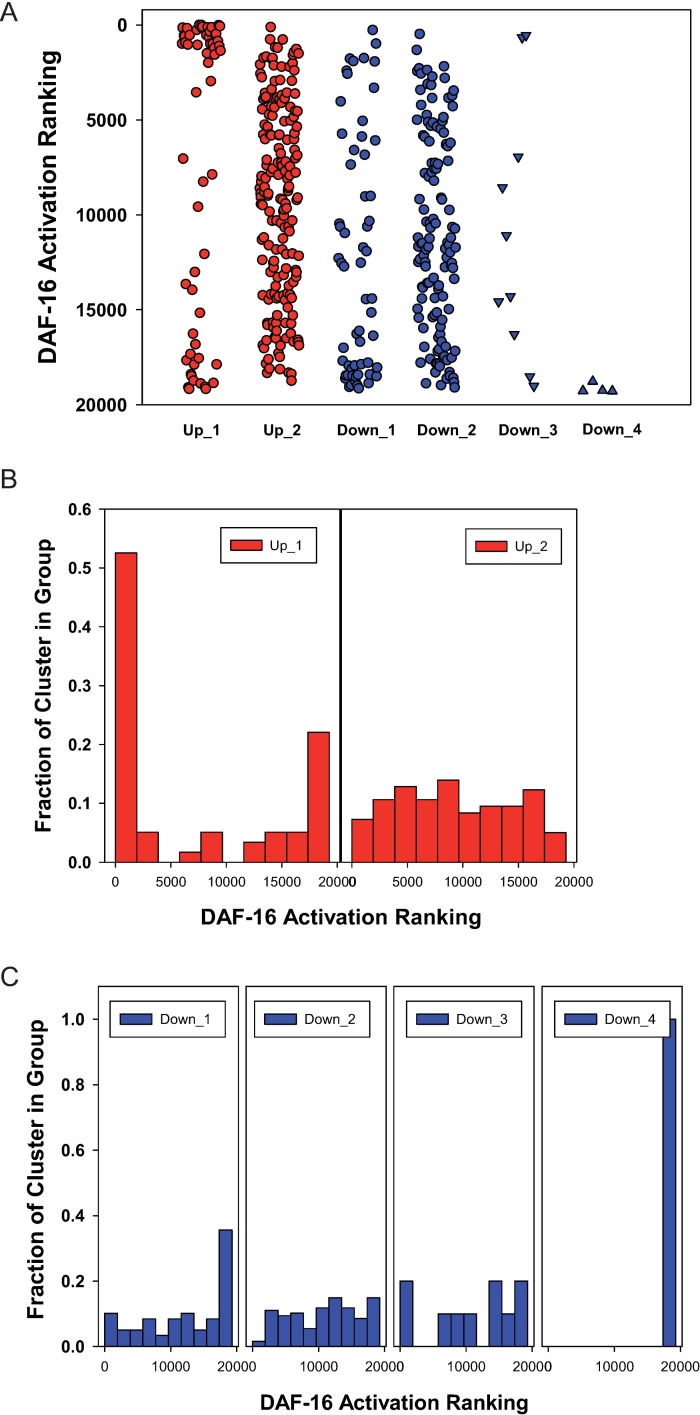
DAF-16 targets and their distribution in HSP90 RNAi induced expression clusters. All the genes distributed into the clusters of Fig 2 are evaluated, whether they are ranked DAF-16 Class I (upregulated) or DAF-16 Class II (downregulated) targets. To this end the ranking was employed from Tepper *et al*. [63]. A) For each cluster the position of the genes is marked by a point at the respective ranking position. The top 1663 genes represent targets considered upregulated by DAF-16; the bottom 1733 genes represent targets downregulated by DAF-16 according to this ranking. B) Histograms for the two clusters *daf-21*Up_1 and *daf-21*Up_2, where the bar on the left side roughly represents the 1663 Class I targets and the bar on the right side the 1733 Class II targets. C) Histograms for the clusters downregulated after Hsp90 RNAi.

As expected, the expression in reporter strains for *skr-5*, *clec-60* and *dod-24* is most affected by Hsp90 RNAi in the intestine, the place where most reactions of the immune response take place. It is interesting that several prominent hits of this response group are regulated by the transcription factor DAF-16, a known regulator of the immune response [60–62]. *skr*-5 and *dod*-24 are direct target genes of DAF-16, along with *hsp*-16.2, *fbxa*-163, C08E8.4, F13D11.4, and Y105C5A.12 [44, 51], which also are among our top induced genes. Given that DAF-16 target genes are well described, we could test whether certain clusters of our networks are strongly enriched for DAF-16 targets, which would suggest that DAF-16 activity is influenced in the Hsp90 depleted nematodes. To this end, we employed a recently published genome-wide ranking of DAF-16 targets, which assigned 1663 Class I targets (upregulated by DAF-16) and 1733 Class II targets (downregulated by DAF-16 and its co-regulator PQM-1) [44, 63]. Using this resource, we determined the ranking number for each clustered gene (Fig 6A, S9 Fig) and tested whether any of our clusters show significant DAF-16 correlation. It is evident that cluster *daf-21*Up_1 in particular contains genes which are regulated by DAF-16 (Fig 6B left plot, S9 Fig). All told, 71% of the genes in this cluster are assigned as DAF-16 targets, which is significantly enriched over the 18% expected by normal distribution. Clusters *daf-21*Down_1 and *daf-21*Down_4 also show enrichment of DAF-16 targets, but they are formed from genes repressed by DAF-16/PQM-1 (Fig 6C, S9 Fig). These data suggest that the DAF-16 pathway is highly active after Hsp90 RNAi. It is interesting in this respect that the induced DAF-16/PQM-1 response differs from the published target ranking, as a group of 12 genes typically repressed by DAF-16/PQM-1 are now activated in *daf-21*Up_1. This may suggest that the balanced regulation between DAF-16 and its transcription suppressing co-regulator PQM-1 could be altered by Hsp90 RNAi, leading to the induction of the innate immune response as observed here.

### Germline-specific markers confirm effects of Hsp90-depletion on events before fertilization

Germline development is disrupted in Hsp90-depleted nematodes. This is evident from the sporadic failure of Hsp90-depleted nematodes to reach fertility and develop correct gonadal or vulval structures [4]. The germline-specific changes were not visible on the proteomic level, likely because most of these proteins are not among the 400 proteins we could quantify. However the transcriptional response does show very consistent genome-wide changes. In cluster *Daf-21*down_1 and *Daf-21*down_2, we find many genes which have a relationship to germline and embryo development. This matches the disruptive influence of Hsp90 RNAi on development. It nevertheless has to be kept in mind that GFP-expression from genomic integrated plasmid arrays may not entirely correlate with the native context of this protein. Thus, a more detailed analysis of the affected cell types would need to involve detection methods for endogenous mRNA or protein, like fluorescence *in situ* hybridization or antibody staining. Despite this the tendency is obvious that most tested target genes from this cluster are coexpressed within the gonads and developing embryos. Thus, reporters for the promoter activity and protein localization of *cav-1*, *zip-8* and *sepa-1* will be helpful to get information on the delayed processes during development. How these changes originate is still enigmatic but the identification of defined developmental alterations related to the observed phenotype could serve as a starting point to study the failures during development after Hsp90-knock down in more detail.

Could the fertility problems also be responsible for the immune response? Several studies have been published about the influence of germline development on the immune response and they suggest that disruption to early germline development can induce the immune response genes by a soma-protective pathway [60, 64, 65]. It remains unclear whether the incomplete embryo development as observed after Hsp90 RNAi can induce the soma-protective immune response. Only in sporadic cases where the gonad arms are obviously shortened by the Hsp90 knock-down are the meiotic germ cells also affected. In our networks, the genes overexpressed after Hsp90 RNAi share very few coexpression relationships with the large gene cluster related to the growth defects. This would imply that the upregulation of these immune response genes usually is not chronologically coupled to the growth defects but is independently regulated instead. To gain more information on this, we individually observed genes which should be upregulated if the soma-protective pathway is active, which would connect the reduction in mitotic germ cells to the activation of the DAF-16 and ATF-7 transcription factors. An important gene, upregulated as part of this pathway is *irg-7*/F40F4.6, which influences the DAF-16 regulated longevity genes and the PMK-1/ATF-7 regulated immunity genes [66]. We see very limited upregulation of *irg-7* in our microarray data (log_2_(DiffExp) = 0.51) and we do not see a systematic influence on PMK-1/ATF-7 targets [38, 67–69] (F08G5.6: log_2_(DiffExp) = 0.14, C09H5.2: log_2_(DiffExp) = 1.03, C17H12.8: log_2_(DiffExp) = 0.25, F56D6.2: log_2_(DiffExp) = -0.14, T24B8.5: log_2_(DiffExp) = -0.99, K08D8.5: log_2_(DiffExp) = -0.08). Given that these genes should be activated via *irg-7* this may imply that the soma-protective route via *irg-7* is not triggered extensively after Hsp90-depletion. While these relationships need to be investigated in further detail, the presented variations provide a starting point to disentangle the complex relationships following the depletion of Hsp90 in *C*. *elegans*.

## Supporting information

S1 Fig Microarray analysis of Hsp90-depleted nematodes. Parameters obtained from the cluster analysis for the top 250 differentially expressed hits are shown for each of the three RNAi experiments, which evaluate the significance of the networks (first replicate: column 1 and 2, second replicate: column 3 and 4, third replicate: column 5 and 6) and the average (column 7 and 8). A) Percentage of hits included in the network for all three experiments. As comparison the results for random gene lists (columns 9 and 10) are depicted. A hit gene is counted as included in the network if a single coexpression connection to any other hit gene is found in the coexpression database. Most of the connections in the random networks are only isolated hit-to-hit pairs. B) Average counts of connections in the network per hit. Two genes will yield many connection counts, if this pair is marked as coexpressed several times in the database. C) CoRegScore for each network to quantify the predictive strength of the network. The score is calculated as described in the Materials and Methods section.
(TIF)

S2 Fig Up-and downregulation of the Hsp90-responsive clusters in the first RNAi experiment. Blue indicates the different levels of downregulation, shadings of red highlight upregulation.
(TIF)

S3 Fig Up-and downregulation of the Hsp90-responsive clusters in the second RNAi experiment. Blue indicates the different levels of downregulation, shadings of red highlight upregulation.
(TIF)

S4 Fig Up-and downregulation of the Hsp90-responsive clusters in the third RNAi experiment. Blue indicates the different levels of downregulation, shadings of red highlight upregulation.
(TIF)

S5 Fig Up-and downregulation of the Hsp90-responsive clusters in the RNAi experiments with depletion of *sams-1*. The three replicates of this experiment were averaged to obtain the depicted expression values. Blue indicates the different levels of downregulation, shadings of red highlight upregulation.
(TIF)

S6 Fig Up-and downregulation of the Hsp90-responsive clusters in the RNAi experiments with depletion of *sbp-1*. All three replicates of this experiment were averaged here. Blue indicates the different levels of downregulation, shadings of red highlight upregulation.
(TIF)

S7 Fig Up-and downregulation of the Hsp90-responsive clusters in the response to *Vibrio cholerae*. Strain VC109, which induces the immune response was compared with VC110, which does not [36]. Blue indicates the different levels of downregulation, shadings of red highlight upregulation. Genes, which were not tested in this microarray experiment, were omitted from the figure.
(TIF)

S8 Fig Up-and downregulation of the Hsp90-responsive clusters in the response to *Pseudomonas aeruginosa* [37]. Blue indicates the different levels of downregulation, shadings of red highlight upregulation. Genes not tested in this microarray experiment were omitted from the figure.
(TIF)

S9 Fig Clustering of DAF-16 targets in the Hsp90-RNAi response network. DAF-16 targets are colored according to the class they were assigned in the genome-wide ranking from Tepper *et al*. [63]. Red nodes indicate Class I targets, which are upregulated by DAF-16. The shadings imply the ranking within this group of 1663 genes, with the most intense red tone marking the strongest DAF-16 targets. Blue indicates Class II targets in this ranking. These genes are downregulated by DAF-16 together with the transcription factor PQM-1. The most intense blue tone indicates the strongest class II target. Genes, which could not be found in this ranking are omitted, the 15890 genes not considered DAF-16 targets by Tepper et al. are white.
(TIF)

S1 Table Proteins with increased levels in Hsp90-depleted nematodes. The proteins listed in this table showed increased levels after Hsp90-RNAi treatment. Protein levels were obtained after comparing the isotope-tagged sample with the non-tagged sample. Averages of two experiments were calculated. A proteins was only included in the final list if several different peptides were quantified for it.
(DOCX)

S2 Table Proteins with reduced level in Hsp90-depleted nematodes. The proteins listed in this table showed reduced levels after Hsp90-RNAi treatment. Protein levels were obtained after comparing the isotope-tagged sample with the non-tagged sample. Averages of two experiments were calculated. A protein was only included in the final list if several different peptides were quantified for it.
(DOCX)
