# Supplementary figures and images for: Hsp90-downregulation influences the heat-shock response, innate immune response and onset of oocyte development in nematodes
Source: PLoS One. 2017 Oct 27;12(10):e0186386. doi: 10.1371/journal.pone.0186386 (PMC5659845; doi:10.1371/journal.pone.0186386)

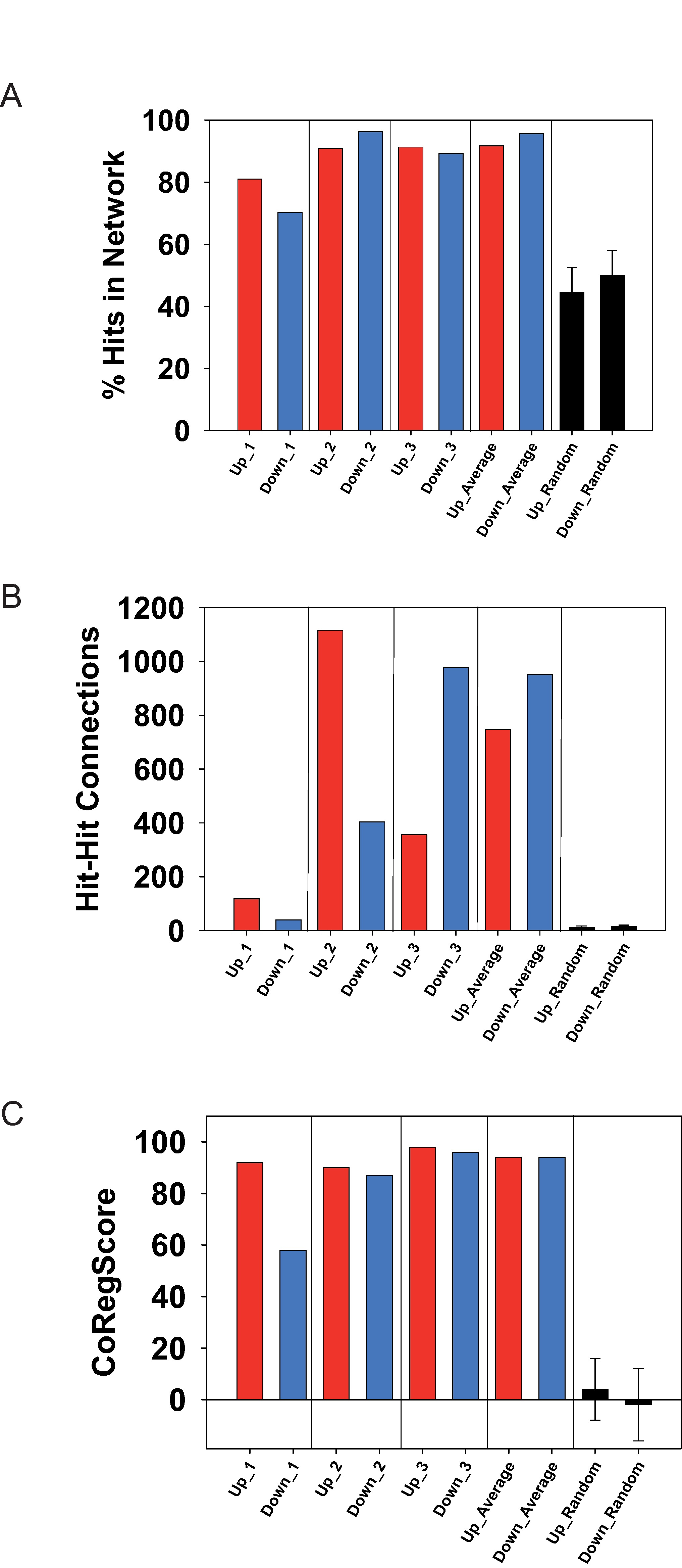

Supplement: S1 Fig — Parameters obtained from the cluster analysis for the top 250 differentially expressed hits are shown for each of the three RNAi experiments, which evaluate the significance of the networks (first replicate: column 1 and 2, second replicate: column 3 and 4, third replicate: column 5 and 6) and the average (column 7 and 8). A) Percentage of hits included in the network for all three experiments. As comparison the results for random gene lists (columns 9 and 10) are depicted. A hit gene is counted as included in the network if a single coexpression connection to any other hit gene is found in the coexpression database. Most of the connections in the random networks are only isolated hit-to-hit pairs. B) Average counts of connections in the network per hit. Two genes will yield many connection counts, if this pair is marked as coexpressed several times in the database. C) CoRegScore for each network to quantify the predictive strength of the network. The score is calculated as described in the Materials and Methods section. (TIF) [file pone.0186386.s001.tif]

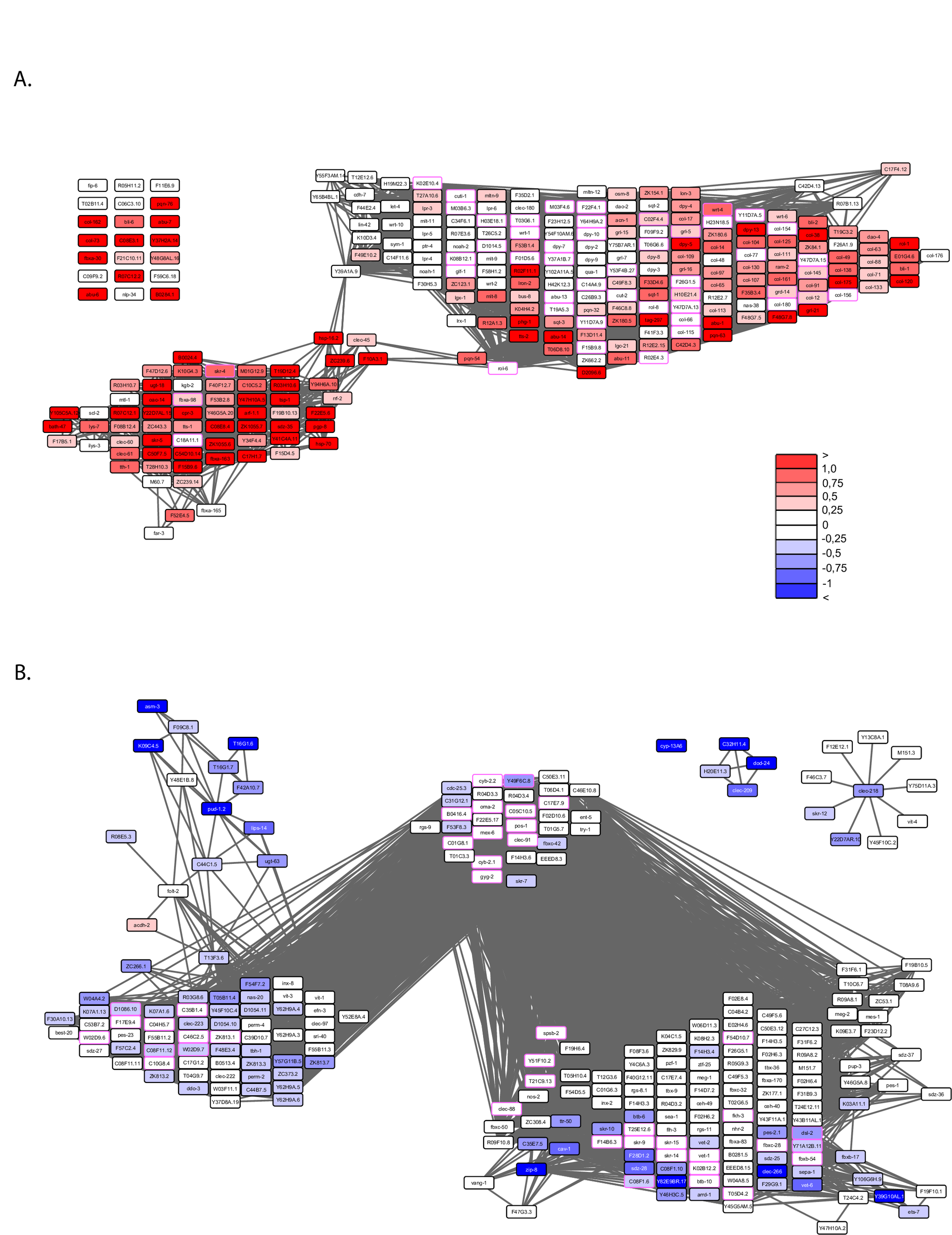

Supplement: S2 Fig — Blue indicates the different levels of downregulation, shadings of red highlight upregulation. (TIF) [file pone.0186386.s002.tif]

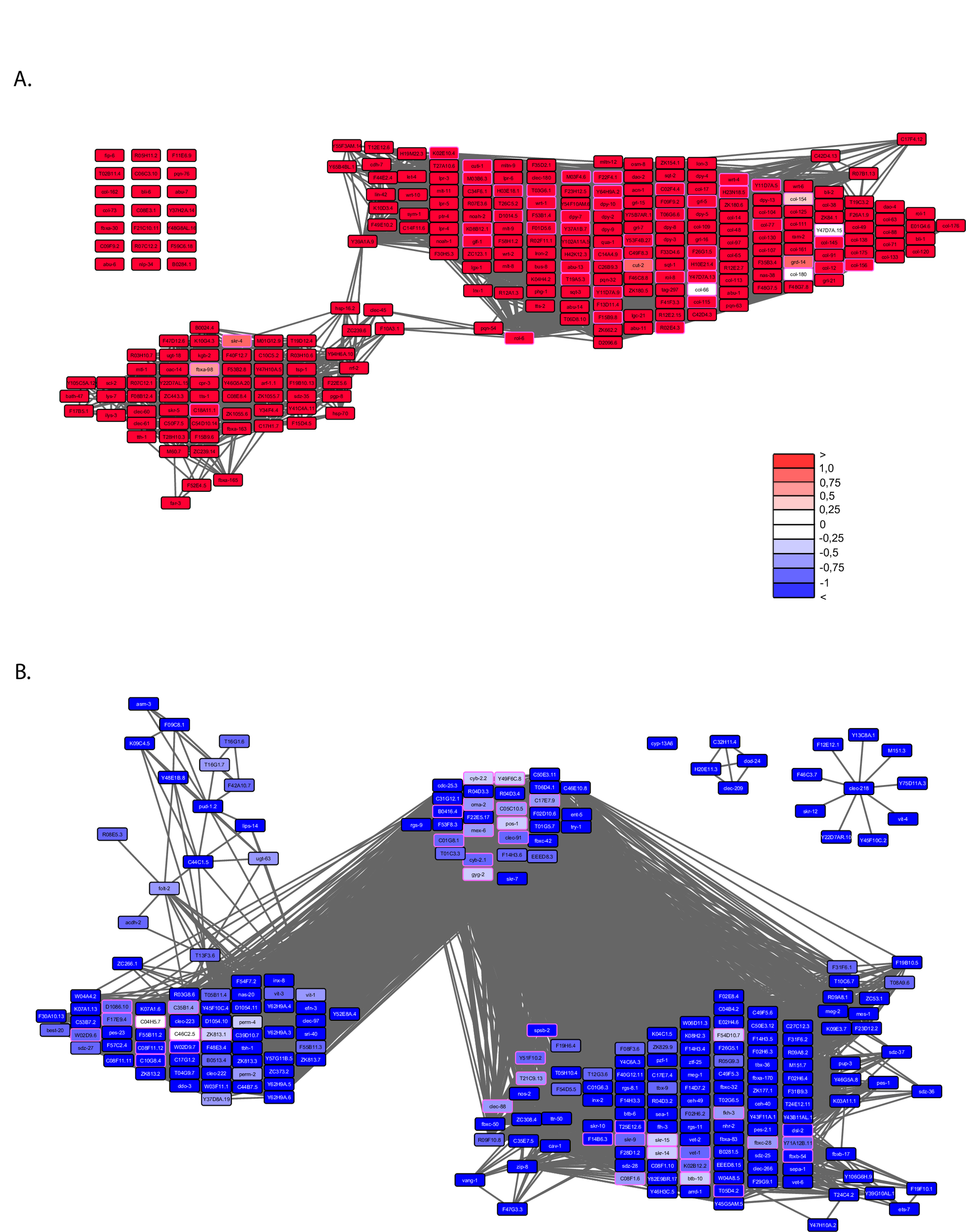

Supplement: S3 Fig — Blue indicates the different levels of downregulation, shadings of red highlight upregulation. (TIF) [file pone.0186386.s003.tif]

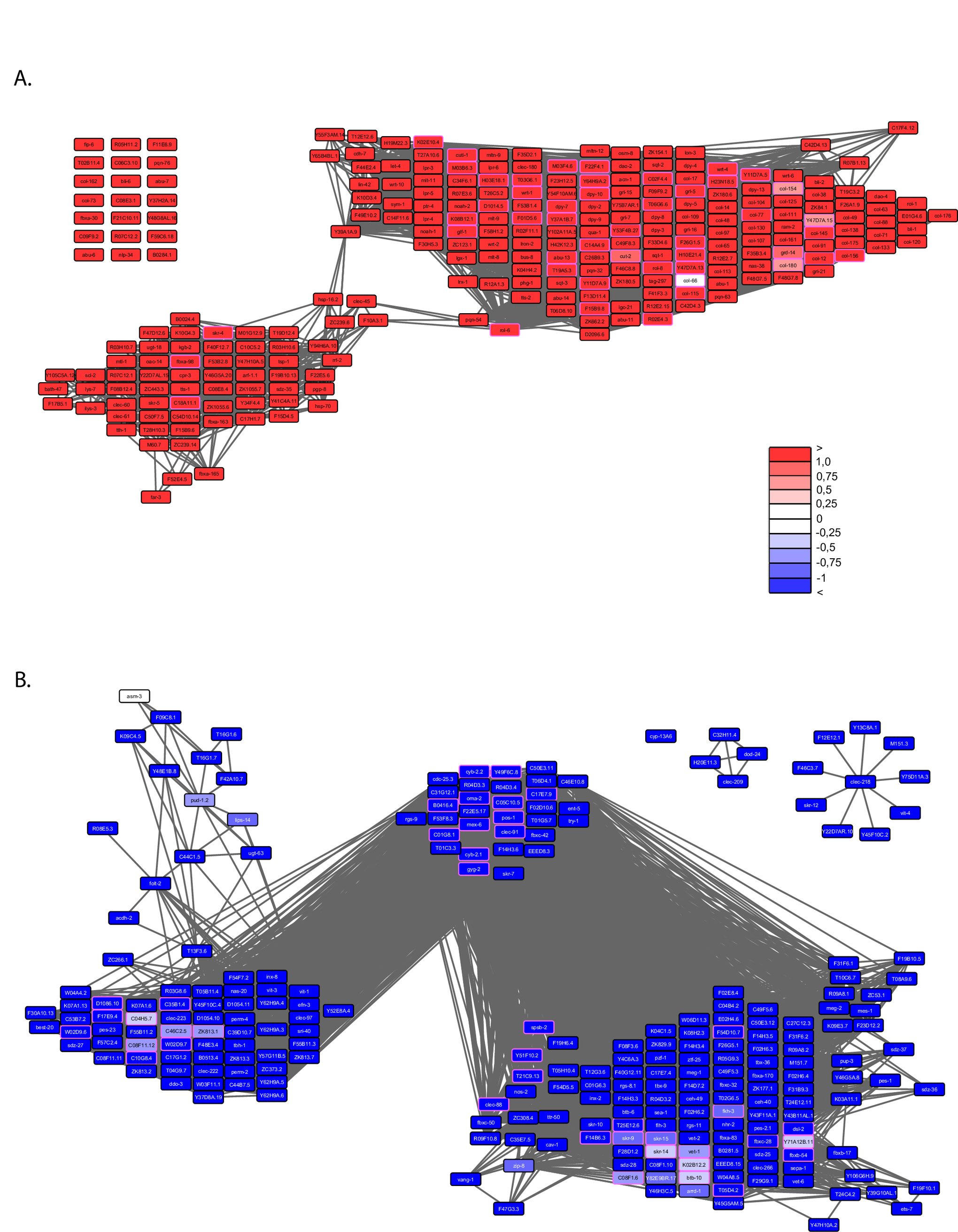

Supplement: S4 Fig — Blue indicates the different levels of downregulation, shadings of red highlight upregulation. (TIF) [file pone.0186386.s004.tif]

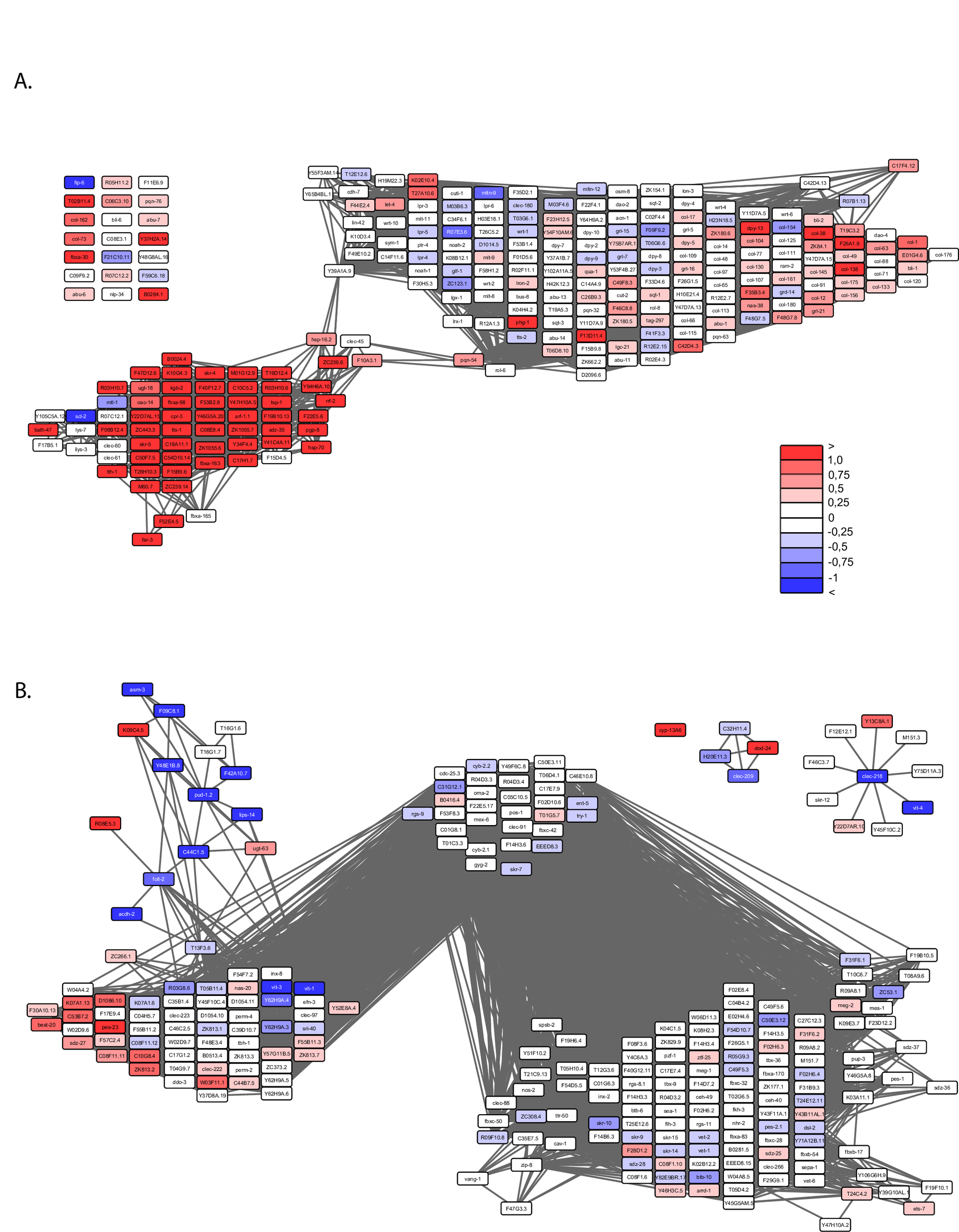

Supplement: S5 Fig — The three replicates of this experiment were averaged to obtain the depicted expression values. Blue indicates the different levels of downregulation, shadings of red highlight upregulation. (TIF) [file pone.0186386.s005.tif]

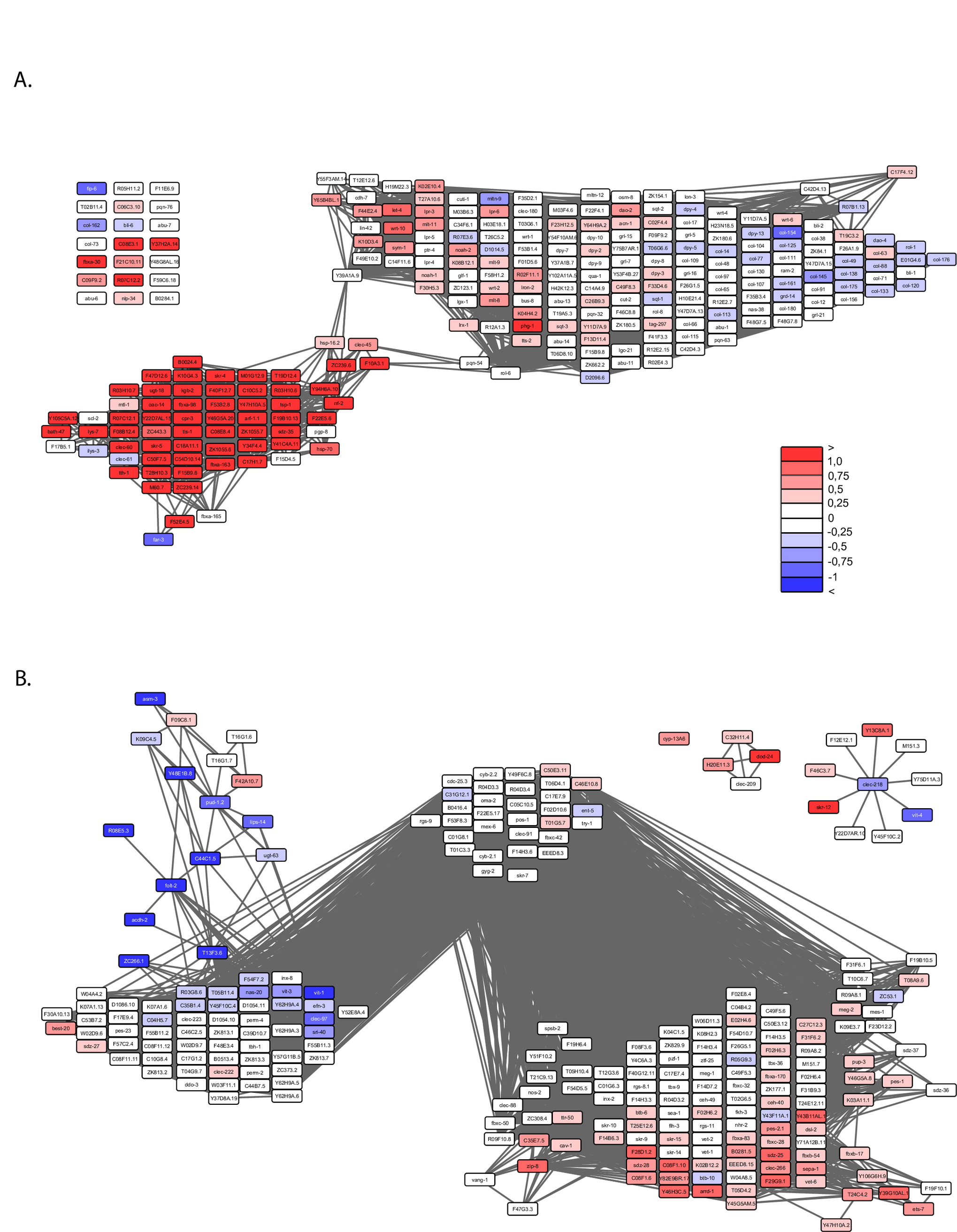

Supplement: S6 Fig — All three replicates of this experiment were averaged here. Blue indicates the different levels of downregulation, shadings of red highlight upregulation. (TIF) [file pone.0186386.s006.tif]

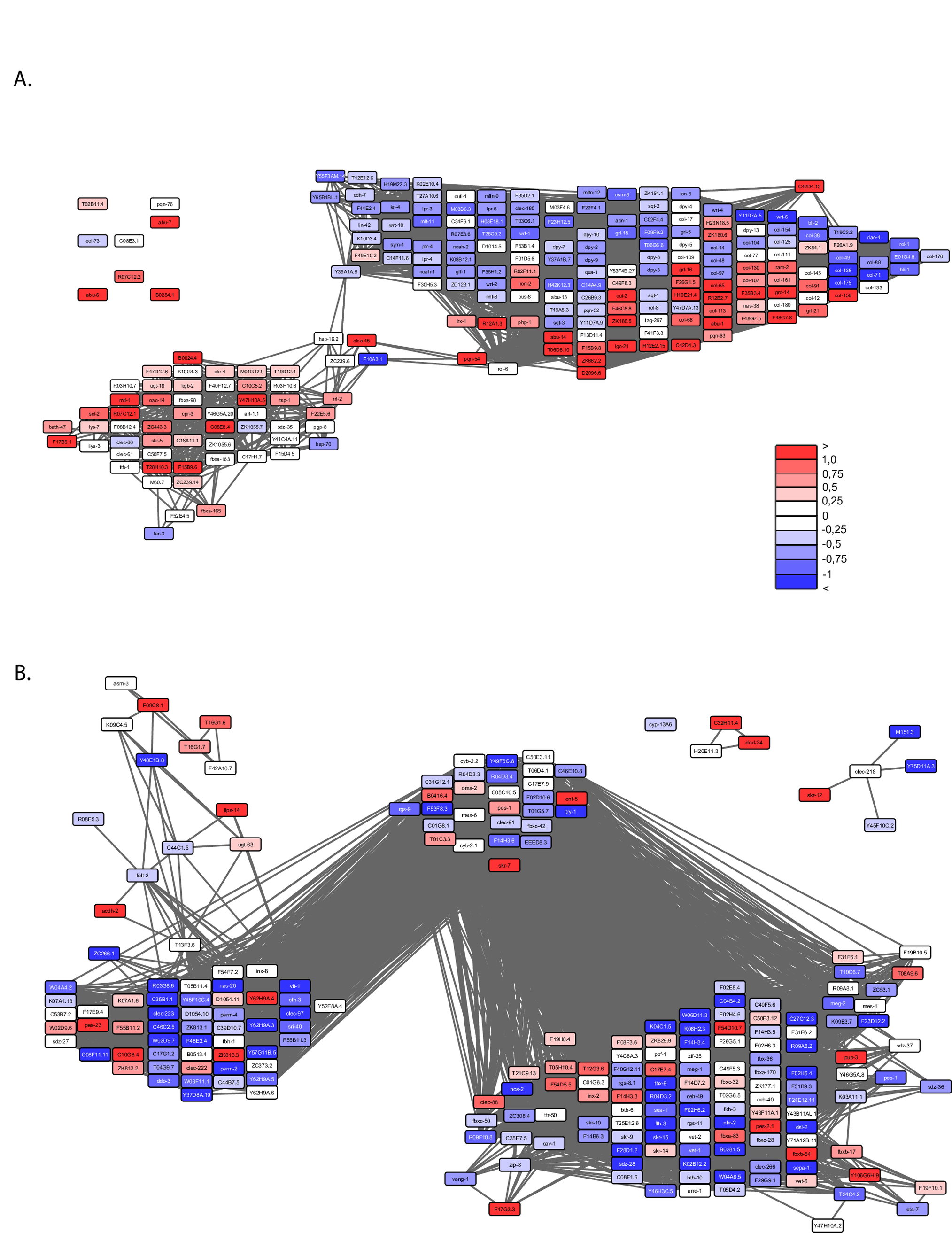

Supplement: S7 Fig — Strain VC109, which induces the immune response was compared with VC110, which does not [36]. Blue indicates the different levels of downregulation, shadings of red highlight upregulation. Genes, which were not tested in this microarray experiment, were omitted from the figure. (TIF) [file pone.0186386.s007.tif]

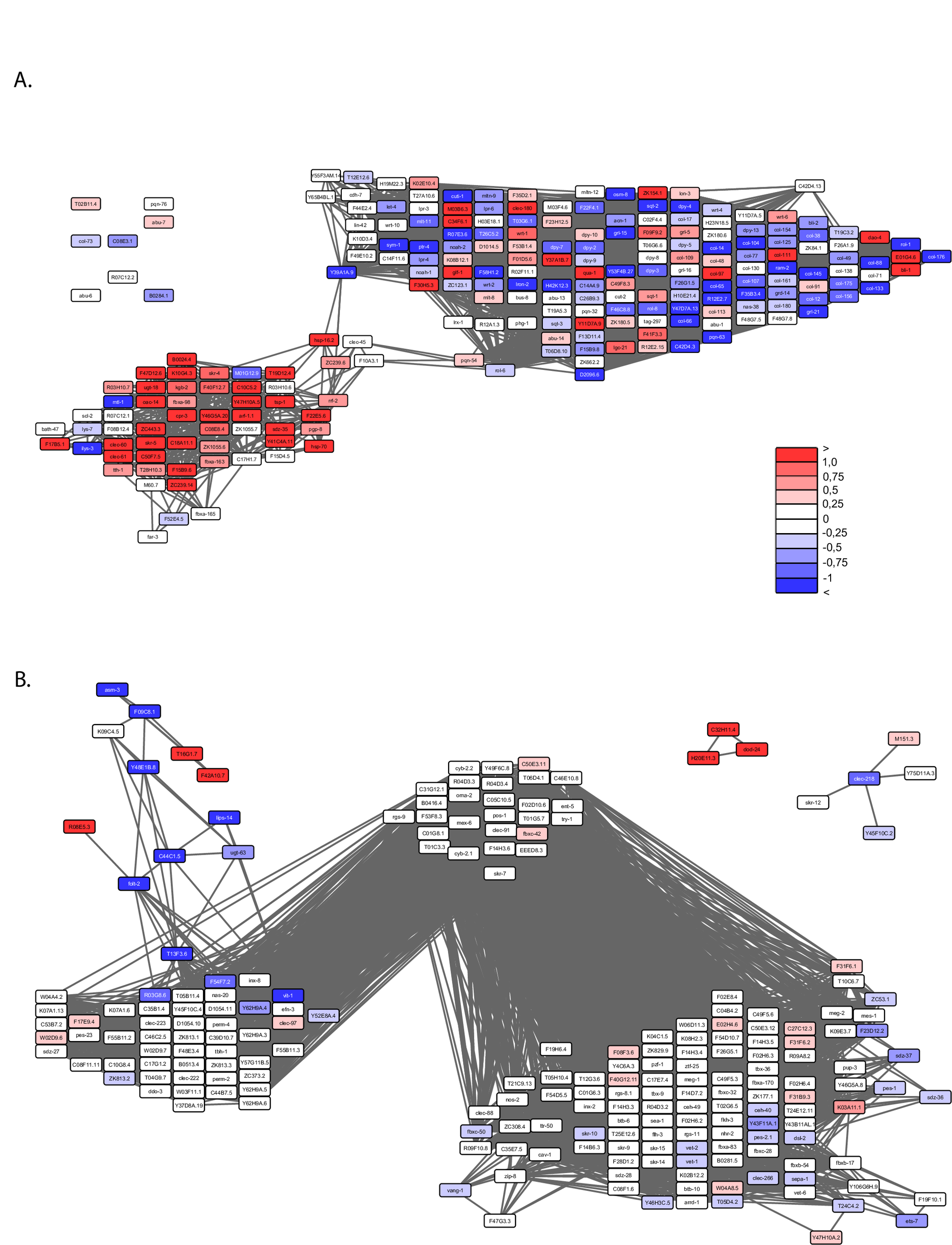

Supplement: S8 Fig — Blue indicates the different levels of downregulation, shadings of red highlight upregulation. Genes not tested in this microarray experiment were omitted from the figure. (TIF) [file pone.0186386.s008.tif]

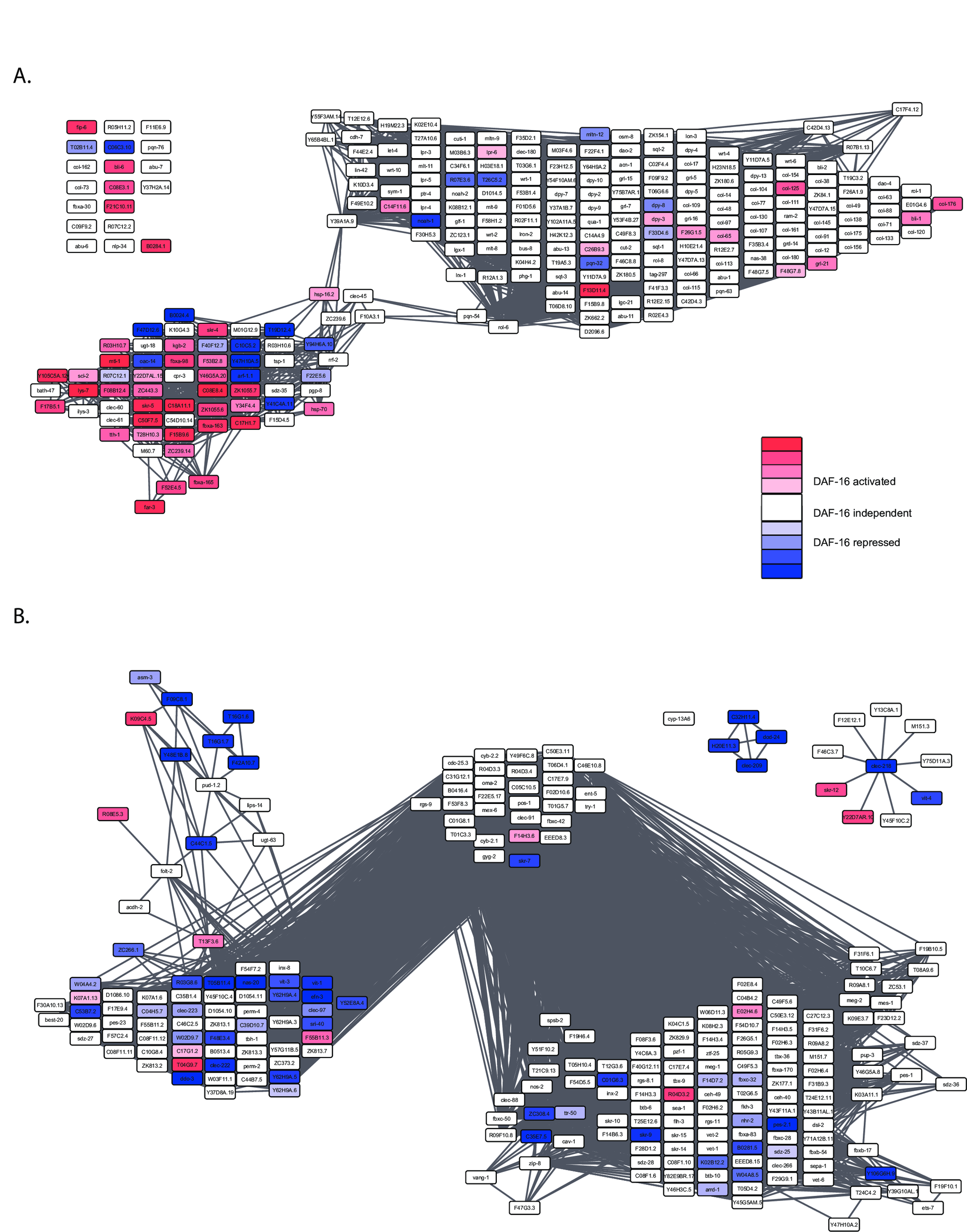

Supplement: S9 Fig — DAF-16 targets are colored according to the class they were assigned in the genome-wide ranking from Tepper et al. [63]. Red nodes indicate Class I targets, which are upregulated by DAF-16. The shadings imply the ranking within this group of 1663 genes, with the most intense red tone marking the strongest DAF-16 targets. Blue indicates Class II targets in this ranking. These genes are downregulated by DAF-16 together with the transcription factor PQM-1. The most intense blue tone indicates the strongest class II target. Genes, which could not be found in this ranking are omitted, the 15890 genes not considered DAF-16 targets by Tepper et al. are white. (TIF) [file pone.0186386.s009.tif]
