# Supplementary material for: Hsp90-downregulation influences the heat-shock response, innate immune response and onset of oocyte development in nematodes
Source: PLoS One. 2017 Oct 27;12(10):e0186386. doi: 10.1371/journal.pone.0186386 (PMC5659845; doi:10.1371/journal.pone.0186386)
Supplement: S1 Table — The proteins listed in this table showed increased levels after Hsp90-RNAi treatment. Protein levels were obtained after comparing the isotope-tagged sample with the non-tagged sample. Averages of two experiments were calculated. A proteins was only included in the final list if several different peptides were quantified for it. (DOCX) [file pone.0186386.s010.docx]

**S1 Table. Proteins with increased levels in Hsp90-depleted nematodes.**

| Protein | Log2 Exp1 | Log2 Exp2 | Ave | STD |
| --- | --- | --- | --- | --- |
| HSP-16.1, 16.2, 16.48 | 3.9 | 4 | 3.95 | 0.07071068 |
| Y94H6A.10 | 3.16 | 3.47 | 3.315 | 0.2192031 |
| Y41C4A.11 | 4 | 2.12 | 3.06 | 1.32936075 |
| UNC-15 | 0.67 | 3.9 | 2.285 | 2.2839549 |
| PUD-2.1 | 1.93 | 1.09 | 1.51 | 0.5939697 |
| UNC-45 | 1.02 | 1.74 | 1.38 | 0.50911688 |
| STI-1 | 1.41 | 1.3 | 1.355 | 0.07778175 |
| F20D1.3 | 0.68 | 1.7 | 1.19 | 0.72124892 |
| C30C11.4 | 1.36 | 0.94 | 1.15 | 0.29698485 |
| DNPP-1 | 1.27 | 0.87 | 1.07 | 0.28284271 |
| CCT-4 | 0.98 | 1.09 | 1.035 | 0.07778175 |
| CCT-7 | 0.98 | 1.08 | 1.03 | 0.07071068 |
| CCT-5 | 0.97 | 1.03 | 1 | 0.04242641 |
| CCT-3 | 0.96 | 1.04 | 1 | 0.05656854 |
| CCT-6 | 0.94 | 1.06 | 1 | 0.08485281 |
| CYC-2 | 0.99 | 1 | 0.995 | 0.00707107 |
| CCT-2 | 0.94 | 1.04 | 0.99 | 0.07071068 |
| HSP-1/HSP-70 | 1.04 | 0.92 | 0.98 | 0.08485281 |
| CCT-8 | 0.94 | 1.01 | 0.975 | 0.04949747 |
| CCT-1 | 0.95 | 0.93 | 0.94 | 0.01414214 |
| FKB-6 | 0.61 | 1.12 | 0.865 | 0.36062446 |
| IDHG-1 | 0.74 | 0.95 | 0.845 | 0.14849242 |
| ASNS-2 | 0.6 | 1.04 | 0.82 | 0.31112698 |
| R05F9.6 | 0.82 | 0.82 | 0.82 | 0 |
| ZC395.10 | 0.94 | 0.6 | 0.77 | 0.24041631 |
| TTLL-12 | 0.82 | 0.7 | 0.76 | 0.08485281 |
| CDC-37 | 0.67 | 0.81 | 0.74 | 0.09899495 |
| F46H5.7 | 0.66 | 0.7 | 0.68 | 0.02828427 |
| SOD-1 | 0.64 | 0.65 | 0.645 | 0.00707107 |
| BAG-1 | 0.61 | 0.67 | 0.64 | 0.04242641 |

The proteins listed in this table showed increased levels after Hsp90-RNAi treatment. Protein levels were obtained after comparing the isotope-tagged sample with the non-tagged sample. Averages of two experiments were calculated. A proteins was only included in the final list if several different peptides were quantified for it.
