# Supplementary material for: Hsp90-downregulation influences the heat-shock response, innate immune response and onset of oocyte development in nematodes
Source: PLoS One. 2017 Oct 27;12(10):e0186386. doi: 10.1371/journal.pone.0186386 (PMC5659845; doi:10.1371/journal.pone.0186386)
Supplement: S2 Table — The proteins listed in this table showed reduced levels after Hsp90-RNAi treatment. Protein levels were obtained after comparing the isotope-tagged sample with the non-tagged sample. Averages of two experiments were calculated. A protein was only included in the final list if several different peptides were quantified for it. (DOCX) [file pone.0186386.s011.docx]

**S2 Table. Proteins with reduced level in Hsp90-depleted nematodes.**

| Protein | Log2 Exp1 | Log2 Exp2 | Ave | STD |
| --- | --- | --- | --- | --- |
| CLEC-4 | -4 | -4 | -4 | 0 |
| CLEC-66 | -3.9 | -4 | -3.95 | 0.07071068 |
| CLEC-83 | -1.59 | -3.24 | -2.415 | 1.16672619 |
| F55G11.4 | -3.69 | -2.94 | -3.315 | 0.53033009 |
| C17H12.8 | -3.89 | -2.65 | -3.27 | 0.87681241 |
| CLEC-65 | -2.51 | -2.6 | -2.555 | 0.06363961 |
| CLEC-186 | -3 | -2.48 | -2.74 | 0.36769553 |
| DAF-21/HSP90 | -2.81 | -2.23 | -2.52 | 0.41012193 |
| ZK6.11 | -1.08 | -2.07 | -1.575 | 0.70003571 |
| CLEC-63 | -1.13 | -1.97 | -1.55 | 0.5939697 |
| ASP-6 | -0.87 | -1.52 | -1.195 | 0.45961941 |
| ASP-2 | -0.83 | -1.5 | -1.165 | 0.47376154 |
| K12H4.7 | -0.75 | -1.28 | -1.015 | 0.37476659 |
| T01D3.6 | -1.31 | -1.24 | -1.275 | 0.04949747 |
| ASP-5 | -0.85 | -1.09 | -0.97 | 0.16970563 |
| ACDH-1 | -0.8 | -1.01 | -0.905 | 0.14849242 |
| T16G12.1 | -0.95 | -1 | -0.975 | 0.03535534 |
| HOE-1 | -0.62 | -0.99 | -0.805 | 0.26162951 |
| ZK228.4 | -1.56 | -0.97 | -1.265 | 0.417193 |
| BCAT-1 | -1.07 | -0.91 | -0.99 | 0.11313708 |
| C49C8.5 | -1.04 | -0.9 | -0.97 | 0.09899495 |
| GLF-1 | -2.19 | -0.81 | -1.5 | 0.97580736 |
| EMB-8 | -1.06 | -0.68 | -0.87 | 0.26870058 |
| Y37A1B.5 | -0.73 | -0.66 | -0.695 | 0.04949747 |
| Y38F1A.6 | -0.7 | -0.66 | -0.68 | 0.02828427 |
| MSRA-1 | -0.75 | -0.65 | -0.7 | 0.07071068 |
| T19D12.4 | -0.78 | -0.6 | -0.69 | 0.12727922 |

The proteins listed in this table showed reduced levels after Hsp90-RNAi treatment. Protein levels were obtained after comparing the isotope-tagged sample with the non-tagged sample. Averages of two experiments were calculated. A protein was only included in the final list if several different peptides were quantified for it.
